# Is the combination of behavioral activation and attention training technique effective to reduce depressive symptomatology? A multiple case study

**DOI:** 10.3389/fpsyg.2022.914094

**Published:** 2022-07-20

**Authors:** Audrey Krings, Marie Geurten, Ecaterina Lazari, Sylvie Blairy

**Affiliations:** ^1^Psychology and Neuroscience of Cognition Research Unit (PsyNCog), Université de Liège, Liège, Belgium; ^2^National Fund for Scientific Research (FRS-FNRS), Psychology of Liège, Liège, Belgium

**Keywords:** multiple baseline, depression, behavioral activation, attention training technique, single-case design

## Abstract

**Background:**

This study tested whether the combination of BATD and Attention Training Technique (ATT) is effective to reduce depressive symptomatology and investigate the mechanisms of action underlying the effectiveness of treatment with a multiple N-of-1 trials.

**Methods:**

Nine adults with depressive symptoms were randomly included in three different combinations of BATD and ATT, concurrent in Condition 1 and sequential in Conditions 2 and 3 (ATT followed by BATD and BATD followed by ATT, respectively). The sequential components allow investigating the specific changes that occur during the two distinct treatment phases. Multiple self-report and pre–post-assessments were conducted on generic mental health measures (depressive symptoms, life functioning, mood, and well-being) and intervention-specific measures (behavioral activation, behavioral avoidance, self-focused attention, cognitive control and rumination), with two-week and three-month follow-up assessments. We also measured treatment adherence with treatment attendance, homework compliance and a clinical interview.

**Results:**

Participants’ attendance, homework compliance and satisfaction were acceptable in the three conditions, with higher adherence in Condition 1 and Condition 3. Eight participants out of nine reported a reduction in depressive symptomatology and five an improvement in well-being. Most of their progress was maintained 2 weeks after the intervention but not 3 months later. Conditions 1 and 2 seemed to be associated with a higher response to generic mental health measures in comparison with Condition 3. The three conditions were not associated with consistent changes in intervention-specific measures, except for rumination with five participants out of nine reporting an improvement in rumination immediately after the intervention and eight participants 2 weeks after the intervention. The concurrent format was associated with a better improvement in rumination immediately after the intervention. No specific changes of self-focused attention and rumination characterized ATT, and no specific changes of behavioral activation, behavioral avoidance and rumination characterized BATD.

**Conclusion:**

Our three interventions were judged acceptable and showed positive short-term benefit for generic mental health measures and rumination maintained 2 weeks later, but not 3 months later. Results suggest that five sessions of concurrent treatment could be a better option than sequential formats. However, our data did not support the specificity of ATT and BATD treatments.

****Clinical Trial Registration:** This trial was previously registered with the ClinicalTrials.gov NCT04595539 registration number and the title “Does Attention Training Technique Enhance the Effectiveness of Behavioral Activation Treatment for Depression: A Multiple Baseline Study.”:**

## Introduction

Depression is one of the most prevalent mental disorders and one of the main causes of disability worldwide (Kessler and Bromet, 2013). Depression is associated with enormous costs at both the individual (e.g., maintaining a household, managing finances, sustaining interpersonal relationships) and societal levels (e.g., health service uptake, productivity losses and lesser efficiency at work) (World Health Organization, 2017). Behavioral Activation Treatment for Depression (BATD) is a psychological treatment that is easy for patients to understand and for practitioners to implement (Richards et al., 2017). It is associated with robust empirical data indicating that it reduces depressive symptoms (Ekers et al., 2014; Cuijpers et al., 2020) and improves well-being (Mazzucchelli et al., 2010) in subclinical and clinical depression. The main goal of BATD is to re-engage people in their lives by increasing the number of positively reinforcing experiences which, in turn, reduce depression (Lejuez et al., 2001, 2011). Previous empirical studies have shown that BATD is supposed to improve behavioral activation (Collado et al., 2014; Dimidjian et al., 2017), and to reduce behavioral avoidance (Chen et al., 2013) and rumination (McIndoo et al., 2016). Moreover, neuronal activation changes in brain regions associated with cognitive control abilities have been found following BATD, suggesting that BATD could impact cognitive control (Dichter et al., 2010).

While BATD is associated with promising therapeutic findings, the magnitude of the effect size on depressive symptoms in comparison with control conditions ranges from low to medium which suggests that there is room for improvement in response to BATD (Cuijpers et al., 2020; Uphoff et al., 2020). One way to improve psychological treatments is to identify the mechanisms of action underlying the effectiveness of treatment in interaction with inter-individual differences (Kazdin, 2007; Cuijpers et al., 2019).

Among the processes targeted by BATD, rumination plays a central role (Manos et al., 2010). However, only few studies have investigated the effect of behavioral activation (BA) on rumination. According to the impaired disengagement hypothesis model of rumination (Koster et al., 2011, 2017), Lemoult and Gotlib model of depression (LeMoult and Gotlib, 2019) and the HEXAGON model or rumination (Watkins and Roberts, 2020), rumination is influenced by multiple factors, including a low level of attention control. Attention control influences rumination by providing the cognitive resources needed to disengage from it. Consequently, it is possible that BA insufficiently affects rumination and the use of a psychological intervention targeting cognitive control resources might be a promising avenue to increase BATD’s effectiveness.

Because attention control resources are not fully under volitional control, these resources need experiential practice to improve, rather than the verbal processes usually employed in psychotherapy (Watkins and Roberts, 2020). The Attentional Training Technique (ATT) is a procedure that aims to strengthen cognitive functions. In this procedure, participants are instructed to focus on auditory stimuli in order to direct their attention away from repetitive negative thoughts including rumination (Wells, 2009). The aim of ATT is to reduce self-focused attention, increase flexible attentional control over information processing, and promote metacognitive awareness to reduce depressive mood (Fergus et al., 2014; Fergus and Wheless, 2018). ATT is a low-attention-demanding task in which cognitive control is needed to inhibit internal intrusive thoughts and focus on the task (Wells, 2009). Reviews have reported that ATT is associated with large effects on anxiety and depression symptoms (Fergus and Bardeen, 2016; Knowles and Wells, 2018). In a single-case series including patients with depression, ATT has also been shown to clinically reduce rumination and self-focused attention (Papageorgiou and Wells, 2000). A reduction in self-focused attention has also been found in non-clinical population (Fergus et al., 2014). These effects on cognitive resources and rumination suggest that ATT is a promising avenue to increase the effectiveness of BATD.

A previous study investigated the combination of cognitive training (including adaptive Paced Auditory Serial-Addition Task-PASAT and ATT) with four sessions of BATD in a clinically depressed sample (Moshier and Otto, 2017). The authors did not find that the adjunction of this training enhanced BATD outcomes, as similar improvements in rumination and depressive symptoms were observed both with and without this training (Moshier and Otto, 2017). However, in that study, only four sessions of cognitive training were administered, which is well below the approximately 10 training sessions recommended in previous research (Koster et al., 2017). Furthermore, the study investigated only one combination of treatments (BATD and cognitive training concurrently), which did not allow the researchers to explore conditions (e.g., cognitive training followed by BATD or vice versa) that might lead to optimization of the efficacy of both cognitive training and BATD. Moreover, participant adherence to the intervention was not assessed, although it is well known to influence treatment response (Cuijpers et al., 2019). Finally, the randomized trial design used did not allow the examination of intra-individual differences throughout the intervention or inter-individual differences in response to treatment. The present study aims to overcome these limitations and investigate whether ATT could enhance the effectiveness of BATD.

This study is a multiple-case study that tested whether combining BATD and ATT is useful to improve generic mental health measures (depressive symptoms, life functioning, mood, and well-being) at post-test and at the two-week and three-month follow-ups. The study also aimed to document which combination of treatments (e.g., a combination of ATT and BATD, ATT followed by BATD, or BATD followed by ATT), if any, produced the best outcome. Moreover, this study aimed to investigate the mechanisms of action underlying the effectiveness of treatment (behavioral activation, behavioral avoidance, and rumination for BATD; self-focused attention, cognitive control, and rumination for ATT).

To do so, we used a multiple-baseline mixed-method case series with multiple baselines across participants, settings and behaviors. This design allows one to capture intra-individual differences with multiple daily evaluations of generic mental health measures and intervention-specific measures, as well as inter-individual differences, which is important in the study of depression – a disorder characterized by considerable heterogeneity regarding the nature of the disturbed psychological processes (Philippot et al., 2018) and symptoms (Fried and Nesse, 2015). This design allowed us to study the variables of interest in relation to the sequential or simultaneous introduction of components of an intervention to explore their individual and combined effects (Krasny-Pacini and Evans, 2018). The sequential components allow investigating the specific changes that occur during the two distinct treatment phases and thus see if the targets of the interventions are indeed modified. In the following sections, the comparisons will be made based on measures taken during a one or two-week baseline. In Condition 1, in which ATT and BATD are concurrent, we expected to observe changes in all targets during the combined intervention. In Conditions 2 and 3 we also expected to observe changes in all targets during the combined intervention. More precisely, in Condition 2, in which ATT is followed by BATD, we expected to observe first a modification of ATT targets (i.e., a reduction in self-focused attention and rumination) and a later enhancement of BATD target processes (i.e., an increase in behavioral activation, and a reduction in behavioral avoidance and rumination). In Condition 3, in which BATD is followed by ATT, we expected the reverse pattern of changes, that is, an increase in behavioral activation, and a reduction in behavioral avoidance and rumination with the introduction of BATD, followed by a later reduction in self-focused attention and rumination with the introduction of ATT.

## Materials and methods

### Design

The multiple-baseline mixed-method case series is a Single-Case Experimental Design (SCED) characterized by repeated assessment of multiple measures of interest. SCED uses multiple measurements to capture intra-individual differences before, during and after the intervention to control for natural fluctuations in the assessed behaviors (Kazdin, 2010). Our study followed an A-B-B′ design that was determined *a priori*. The length of the baseline phase, (A) differed between participants (one or two-week baseline). The length of the intervention phase, (B) differed between participants, depending on the condition in which they were randomly included (5 weeks for ATT + BATD; and 7 weeks for ATT-BATD and BATD-ATT). Phase B′ was a two-week follow-up phase. Throughout all phases, participants completed daily measures in a booklet. A high standard SCED design should include a minimum of three replications of the intervention to demonstrate its effect (Kratochwill et al., 2013; Krasny-Pacini and Evans, 2018). Consequently, this study needed a minimum of nine participants (three per condition).

We also collected standardized measures four times during the study protocol (i.e., at pre-treatment, immediately after treatment, at the two-week follow-up and at the three-month follow-up). The timing of these four assessments was determined *a priori* and designed to provide an overall context to help us interpret the daily measure outcomes. The four standardized pre- and post-assessments were conducted by the second author, an external clinician psychologist blind to patient condition and objectives of the study, to minimize the risk of bias due to different roles (therapeutic and evaluative).

### Participants

#### Selection criteria

Regarding inclusion criteria, participants had to be aged between 18 and 65 years, have a good knowledge of French, and have at least a medium level of depressive symptoms (i.e., a score of at least 12 on the Beck Depression Inventory – II). The cut-off applied was based on the one provided by the French validation where a score of at least 12 is considered as depression (Beck et al., 1996; Centre de Psychologie appliquée, 1996). Participants meeting the following criteria were excluded from the procedure: a history of psychotic, bipolar or, neurological disorder; an alcohol/substance dependence other than tobacco in the past 6 months; a concurrent additional psychotherapy; acute suicidal ideation; or a significant change in medication within 1 month prior to baseline assessment. We also excluded participants with severe organic illness (e.g., cancer) as the intervention is not designed to target these specific issues. Finally, we excluded participants who reported modifications in medication intake that could influence our findings throughout the research study.

#### Participant’s demographic and clinical characteristics

Participants are self-referrals from the general population with depressive symptoms. None of the participants followed a concurrent additional psychotherapy. Participants’ ages ranged between 23 and 51 years; there were eight women and one man. Four participants were single and five were in relationships; three of them had children living at home. Eight participants were Belgian and one was Vietnamese. Five participants had full-time paid jobs, three were students and one was unemployed. Four participants reported a low level of depressive symptoms, four reported a medium level of depressive symptoms and one reported a high level of depressive symptoms. Seven participants reported a current major depressive episode and four of them reported low suicidal ideation. Six participants had experienced several past depressive episodes. Of the nine participants, one also suffered from agoraphobia and social phobia. None of the participants was regularly taking anti-depressants or anxiolytic medication. Table 1 presents the participants’ demographic and clinical characteristics.

**Table 1:** Participants’ demographic and clinical characteristics.

Participant	Condition	Age	Gender	Relationship status (number of children)	Origin	Education level	Previous therapy	Employment	Anxiety disorders	MDD	BDI-II scores	Number of past EDM
*S01*	*1*	*23*	*F*	Single	Belgian	LCHE	C	S			13	One
*S05*		*28*	*F*	In relation.	Belgian	SCHE	LI-CBT	S	Agoraphobia Social phob.	MDD L-SI	26	Several
*S08*		*44*	*F*	In relation. (3)	Belgian	SCHE	None	E			25	
*S02*	*2*	*51*	*F*	In relation. (1)	Belgian	College	LI-CBT	E		MDD	18	Several
*S06*		*33*	*F*	In relation. (2)	Belgian	SCHE	C + MBCT LI-CBT	E		MDD L-SI	25	Several
*S09*		*27*	*M*	Single	Vietnamese	SCHE	C	S		MDD	33	Several
*S04*	*3*	*25*	*F*	Single	Belgian	SCHE	C	E		MDD L-SI	22	Several
*S07*		*23*	*F*	Single	Belgian	LCHE	C	U		MDD L-SI	13	
*S10*		*25*	*F*	In relation.	Belgian	HE- LT	None	E		MDD	11	

F, Female; M, Male; In relation., In relationship; SCHE, Short-Course Higher Education; LCHE, Long-Course Higher Education; Previous Therapy: C, Counselling; MBCT, Mindfulness-Based Cognitive Therapy; LI-CBT, Low-Intensity Cognitive Behavioral Therapy; U, Unemployed; E, Employed; S, Student; Social phob., Social Phobia; MDD, Major Depressive Disorder; L-SI, Light Suicidal Ideation; BDI-II scores, Beck Depression Inventory–Second Edition scores.

### Procedure

We posted paper advertisements at the university and digital advertisements in the authors’ social networks to recruit participants. Then, we used a two-phase recruitment protocol. First, the first author conducted a phone interview with interested candidates to provide practical information and screen for eligibility. If a candidate met the inclusion criteria, the first author reassessed his/her eligibility during a pre-clinical interview in order to investigate the person’s level of depressive symptoms, medical history, and complaints.

**Figure 1:**
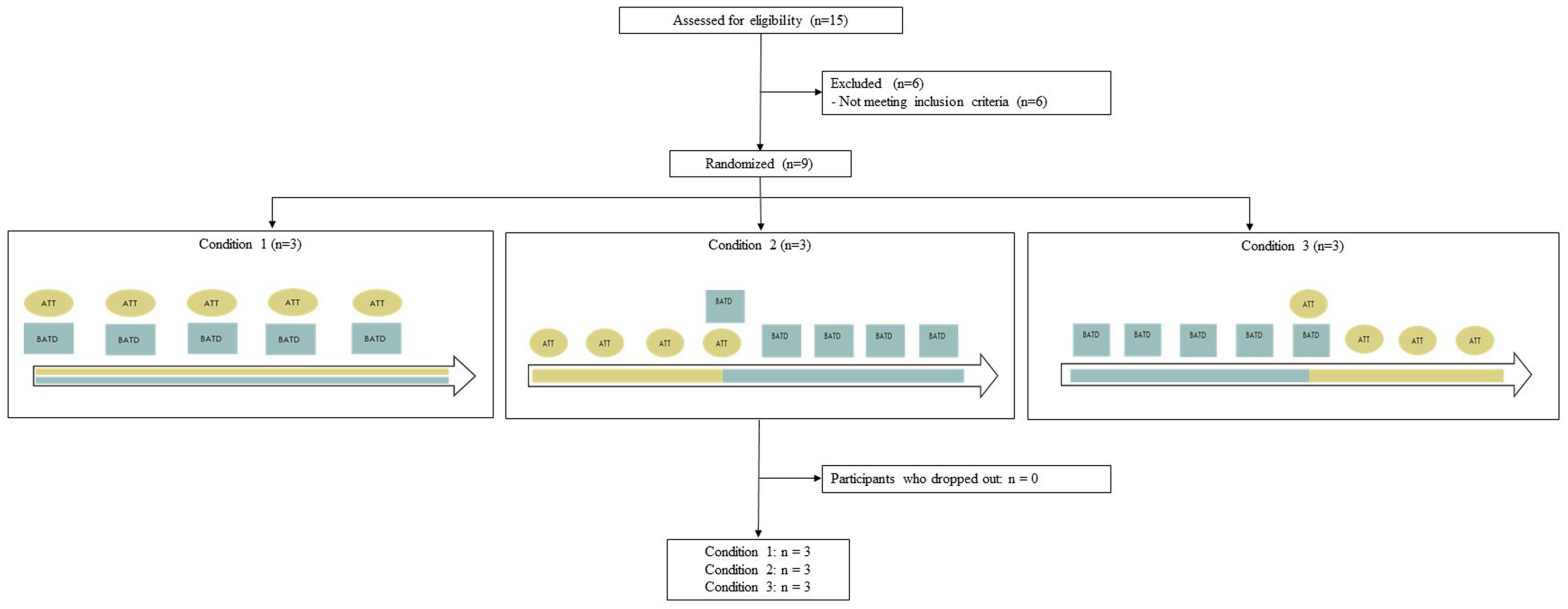
Enrollment chart.

Following confirmation of eligibility, we randomized participants to one of three format conditions. The length of the baseline phase was 2 weeks for the first 5 participants and 1 week for the last 4.^1^ After the baseline phase, participants were invited to come back to the clinic, where they completed a pre-assessment (T0) with an external evaluator and then started one of the three intervention format conditions (Phase B). After the intervention, participants completed a post-assessment evaluation (T1). Multiple measures continued for 2 weeks post-treatment (Phase B′). After this two-week period, participants completed a second post-assessment evaluation (T2) and a third assessment 3 months after the end of the intervention (T3). Figure 1 represents the general procedure.

**Figure 2:**
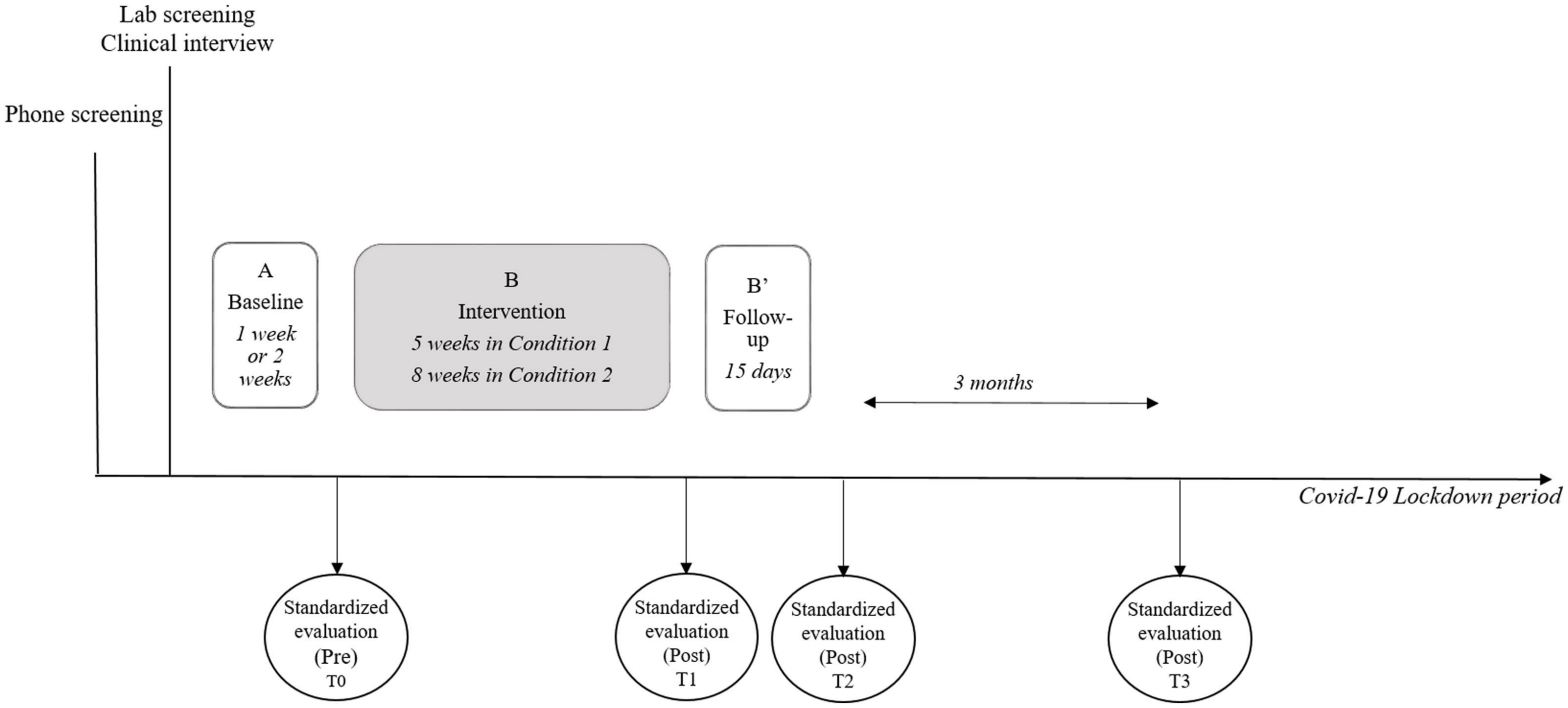
General procedure.

Three conditions were included: a first combining ATT and BATD simultaneously, a second where ATT was followed by BATD, and a third where BATD was followed by ATT. Condition 1 was spread over 5 weeks with 52 h laboratory sessions (1 h of ATT and 1 h of BATD). Additional ATT sessions had to be carried out at home between the laboratory sessions. Condition 2 was spread over 8 weeks, with eight laboratory sessions: 71 h sessions and 12 h session (which included 1 h of ATT followed by 1 h of BATD). Six ATT sessions were prescribed at home. Condition 3 was spread over 8 weeks, with eight laboratory sessions: 71 h sessions and 12 h session (which included 1 h of BATD, followed by 1 h of ATT). Six ATT sessions were prescribed at home. Schemas depicting the three format conditions are presented in the enrollment chart (see Figure 2).

Based on the advertisements, the first author phoned the first 15 interested people. Two candidates were excluded because of a concurrent additional psychotherapy, one because of a significant change in medication within 1 month prior to the baseline, and one because of a recent trauma. Thus, 11 participants met the inclusion criteria and went on to the pre-clinical interview. During the pre-clinical interview two participants were excluded because of a low level of depressive symptoms. The final participants were nine individuals who met the inclusion criteria. Figure 2 represents enrollment charts.

The study was conducted in Belgium during the second wave of the COVID-19 pandemic (i.e., from October 2020 to February 2021). Patients and therapist met in a consultation room of the CPLU – a private clinic located at Université de Liège – or *via* videoconference appointment when face-to-face meetings were not allowed due to the pandemic. The inclusion of participants was non-concurrent (participants started the protocol between October 13, 2020, and October 21, 2020). This trial was previously registered on clinicaltrials.gov.^2^ All participants gave their written informed consent. The order of the questionnaires remained the same for each participant. The Ethics Committee of Université de Liège^3^ approved the study, which was conducted according to the Declaration of Helsinki.

### Measures

We selected the measures in line with treatment rationales, the results of empirical studies and the personal case formulation of each participant.

#### Treatment adherence and satisfaction

As a measure of treatment adherence, we first recorded the session attendance and homework assignments. We conducted a descriptive (non-rated) clinical interview focusing on the patient’s satisfaction with the organization, with the content, and with the therapists.

#### Multiple measurements

Generic mental health measures and intervention-specific measures were included in the multiple measurements. Common (all participants had the same) and personal (specific to participants) measures were collected daily in a booklet in which items were accompanied by a Visual Analog Scale (VAS) from 0 to 10. Regarding measures that were common to all participants, they were asked to estimate their level of behavioral activation, behavioral avoidance, self-focused attention, rumination, and general mood during the day. Common measures were selected based on standardized scales and presented to the participants during the clinical interview to ensure that the construct assessed by the item was clear and validated by the participant (items are presented in the Supplementary Table S1). All common measures were validated by 15 experts (psychology researchers, and clinical psychologists at Liege University) who were asked to rate the validity of the measures (e.g., content validity).

Personal measures, on the other hand, were two personal depressive symptoms and two personal areas of functioning impairments. We used an idiographic approach to measure depressive symptoms and functioning impairment given the heterogeneity of depressive symptoms and associated disturbances that characterizes depression. Symptoms were selected by participants from a list of items containing symptoms of depression, and functioning impairments were selected by participants from a list of six areas of life functioning (i.e., households, work/school, social, professional, hobbies, and relationships). All measures were selected with the therapist during the clinical interview. For each item, a higher score (i.e., placing the cursor closer to the right side of the VAS) indicated higher frequency/intensity of the phenomenon.

#### Pre–post-measurements

Before the intervention, we used a sociodemographic questionnaire to register participants’ characteristics and a French version of The Mini-International Neuropsychiatric Interview (MINI) to assess current mental disorders (Lecrubier et al., 1997). The modules on anorexia, bulimia, and antisocial personality disorder were not used, as they are of limited interest for this study.^4^

For generic mental health measures, we used the Beck Depression Inventory – Second Edition (BDI-II) to assess the severity of depressive symptoms (Beck et al., 1996; Centre de Psychologie appliquée, 1996). In the French version, a score between 12 and 19 is considered as mild depressive symptomatology, a score between 20 and 27 is considered as moderate depressive symptomatology and a score above 28 is considered as severe depressive symptomatology (Centre de Psychologie appliquée, 1996). We also used the Work/School Impairment and Social Impairment subscales of the Behavioral Activation for Depression Scale (BADS) to assess work and social functioning disturbances (Kanter et al., 2007; Krings et al., 2021). We used the Warwick-Edinburgh Mental Well-Being Scale (WEMWBS) to assess well-being (Tennant et al., 2007; Trousselard et al., 2016). Higher scores indicate higher depressive symptoms, work and social impairment, and well-being, respectively.

For intervention’s-specific measures, we used the Activation and Behavioral Avoidance subscales of the BADS to assess behavioral activation and behavioral avoidance (Kanter et al., 2007; Krings et al., 2021). We also used the Abstract Evaluative mode of Repetitive Thinking subscale (AERT) of the Repetitive Thinking Mode Questionnaire (RTMQ) to assess rumination, characterized by thoughts at an abstract, over-general level that address the causes and consequences of one’s mood or condition.^5^ In addition, we used the Internally oriented Attention Subscale (IAS) and the Externally oriented Attention Subscale (EAS) of the Attentional Style Questionnaire (ASQ) to assess self-report attentional control resources (Van Calster et al., 2018). These subscales measure an individual’s capacity to maintain attention on task-related stimuli and not be distracted by internal or external interfering stimuli. Finally, a computerized version of the Paced Auditory Serial-Addition Task (PASAT) was used as a measure of participants’ updating abilities reflecting working memory ability (Gronwall, 1977). In this task, 60 numbers (from 1 to 9) were presented successively. Participants were asked to add each number to the preceding one, which prompted them to update their working memory. The task was divided into four trials that differed in the speed with which the numbers were presented. For each variable, higher scores indicated higher behavioral activation, behavioral avoidance, rumination, attentional resources more easily captured by internal and external stimuli, and cognitive control resources, respectively.

We used the validated French version of all those scales. Number of items, range, fidelity index, mean and standard deviation in general population sample of all those scales are reported in Table 2.

**Table 2:** Fidelity index, mean and standard deviation of standardized subscales.

Measures										
	Behavioral Activation	Behavioral Avoidance	Attentional control	Attentional control	Attentional control	Rumination	Depression	Work/School impairment	Social impairment	Well-being
Scale	BADS	BADS	ASQ	ASQ	PASAT	RTMQ	BDI-II	BADS	BADS	WEMWBS
Subscale (number of items)	Behavioral Activation subscale (7 items)	Behavioral Avoidance subscale (5 items)	Internally oriented Attention Subscale (IAS) (7 items)	Externally oriented Attention Subscale (EAS) (5 items)	- (60 trials)	Abstract Evaluative mode of Repetitive Thinking subscale (AERT) (6 items)	- (21 items)	Work/School Impairment subscale (5 items)	Social Impairment subscale (5 items)	- (14 items)
Range (min-max)	0–42	0–30	7–42	5–30	0–60	6–24	0–63	0–30	0–30	14–70
Fidelity index	*ω* = 0.83	*ω* = 0.85	*α* = 0.79	*α* = 0.76	split half reliability =0.89	*α* = 0.78, ICC = 0.92	*α* = 0.86	*ω* = 0.84	*ω* = 0.83	*α* = from 0.85 to 0.89 (M^*^)
*General population*										
Mean	22.75	7.39	26.42	17.85	50.83	13.52	10.02	10,74	4,36	51.68 (M^*^)
Standard deviation	8.75	6.92	5.99	4.86	9.02	4.85	7.36	7.25	5.72	7.03 (M^*^)
*n*	409	409	111	111	520	138	520	409	409	394
Sources	Krings et al. (2021)		Van Calster et al. (2018)		Krings et al. (2022)	See Footnote 5	Krings et al. (2022)	Krings et al. (2021)		Trousselard et al. (2016)

BADS, Behavioral Activation for Depression Scale; SBI, Savoring Belief Inventory; ASQ, Attentional Style Questionnaire; PASAT, Paced Auditory Serial-Addition Task; RTMQ, Repetitive Thinking Mode Questionnaire; BDI-II, Beck Depression Inventory–Second Edition; WEMWBS, Warwick-Edinburgh Mental Well-Being Scale; ω, Omega; α, Cronbach’s α. M^*^ = Mean computed from student and worker scores.

### Interventions

The protocol used was adapted from the 10-session program based on the *Brief Behavioral Activation Treatment for Depression – Revised Treatment Manual* developed by Lejuez et al. (2001). The intervention was shortened from the minimum of 10 sessions originally proposed by Lejuez et al. (2001). This shortened version was proposed because more recent studies have found that the number of sessions is not a significant moderator of treatment effect (Simmonds-Buckley et al., 2019) and that sudden gains often occur before the fourth session (Blairy et al., 2020). The five-session BATD included the development of a shared formulation, psychoeducation, self-monitoring of daily activities, identifying “depressed behaviors,” developing alternative goal-oriented behaviors, scheduling goal-directed activities, and problem-solving concerning difficulties implementing scheduled activities. The treatment manual is available from the first author on request.

Wells’s Attention Training Technique is a task designed to train selective attention to specific information by teaching individuals to attend to multiple external auditory sources (Papageorgiou and Wells, 2000). Each ATT exercise progressed through stages that trained three different functions. First, there was a six-minute selective attention phase in which participants had to focus their attention on one sound at a time (for 15 s) following the therapist’s instructions. Second, in a six-minute flexibility phase, participants had to disengage their attention from one sound and focus on another sound every 10 s, following the therapist’s instructions, with a speed that increased as the exercises progressed. The third phase was a three-minute divided attention phase in which participants had to count and listen to all sounds simultaneously. One ATT training session took approximately 15 min. We used six different audio-recorded exercises during the therapy, each composed of seven sounds.

As frequently recommended in ATT, the first laboratory ATT session included psychoeducation and a discussion of rumination (e.g., controllability, usefulness) to understand the rationale of treatment. Furthermore, each ATT exercises were also accompanied with a self-report evaluation of self-focus attention before and after the auditory exercises. Ten sessions were prescribed, including five in the laboratory and five at home in Condition 1, and four sessions in the laboratory and six at home in Conditions 2 and 3. At home, we instructed participants to sit in a quiet room and to perform the audio-recorded exercises provided by the therapist (without additional exercises).

The first author (AK), who conducted the interventions, is a clinical psychologist/psychotherapist specializing in cognitive-behavioral therapy under the weekly supervision of the last author, who is an experienced clinical psychologist, psychotherapist and supervisor. To provide ATT, the therapist was supervised by an expert in this technique (M.-N. Levaux).^6^

### Statistical analysis plan

For multiple measurements, we first followed the visual analysis guidelines (Kazdin, 2010) and computed the mean as an index of central tendency, the standard deviation as an index of variability, and the least squares regression as an index of trend. Additionally, to reflect the effect size, we computed the Tau for non-overlap with baseline trend control indices (Tau-U) (Bulté and Onghena, 2013). Tau-U indices measure the difference between phases of treatment by controlling for the baseline trend (Parker et al., 2011). Tau-U was computed online on the website http://www.singlecaseresearch.org (Vannest et al., 2016). In addition, we computed a Cohen *d* score to reflect the effect size of change between mean phases. Cohen *d* scores between 0.20 and 0.50 are considered low, scores between 0.50 and 0.80 are considered medium, and scores of 0.80 or higher are considered as large (Cohen, 1977).

For pre–post-measures, we computed a change score assessing the proportion of individuals showing reliable change (RC) at each post-treatment assessment time, relative to pre-treatment levels. RC allowed us to rule out the possibility that a difference between two scores for a given individual was due to a measurement error rather than to the intervention (Jacobson and Truax, 1991). We focused on both improvement and deterioration to identify benefits and harm.

Following recommendations on research transparency and replicability, de-identified data can be downloaded on the Open Science Framework link: https://osf.io/zcpvf/.

## Results

### Treatment adherence analysis

#### Session attendance and homework compliance

Session attendance and homework compliance are presented in Table 3. All participants completed all sessions but the therapist had to reschedule six sessions because of cancellation, which was usually related to the COVID-19 pandemic. Two participants (S01 and S02) took all the therapy sessions *via* videoconference.^7^ The homework completion rate seems to be higher in Conditions 1 and 3 in comparison with Condition 2.

**Table 3:** Sessions attendance and homework compliance.

		Condition 1			Condition 2			Condition 3		
		S01	S05	S08	S02	S06	S09	S04	S07	S10
Laboratory treatment sessions		0/5	5/5	4/5	0/8	7/8	8/8	5/8	5/8	7/8
Video treatment sessions		5/5	0/5	1/5	8/8	1/8	0/8	3/8	3/8	1/8
Laboratory pre–post-assessments		3/4	2/4	4/4	2/4	1/4	1/4	0/4	2/4	2/4
Video pre–post-assessments		1/4	2/4	0/4	2/4	3/4	3/4	4/4	2/4	2/4
Rescheduled sessions		0	0	2^1^	2^2^	0	1^3^	1^4^	0	0
Activity monitoring (%)		> 70	> 70	> 70	< 30	< 30	< 50	< 50	> 70	> 70
Activity completion (%)		> 50	> 70	> 70	< 30	> 70	< 50	< 50	> 70	> 70
Number of ATT exercises completed at home		4/5	4/5	7/5	1/6	3/6	5/6	2/6	6/6	4/6
Number of ATT exercises completed in the Laboratory		5/5	4/5^*^	5/5	3/4^*^	4/4	3/4^*^	4/4	4/4	4/4
Daily measures completed (%)	Baseline	85.71	66.67	50	93.75	20	75	64.29	87.5	87.5
	Intervention	58.62	71.43	71.43	57.45	48	66.67	53.06	100	95.83
	Follow-up	NA	99.33	100	66.67	44.44	42.11	NA	100	95

Laboratory sessions = Laboratory sessions at Université de Liège; NA = missing data.

* no ATT exercise because the participant arrived late.

1 Quarantining.

2 Felt tired and overburdened.

3 Felt sick.

4 Felt sick because of COVID-19.

#### Clinical interview

All participants were satisfied with the material conditions, but three complained about the poor quality of some audio-recorded exercises. Regarding the format of Condition 1, two of the three participants reported that 2 h was a bit long and very tiring. In Conditions 2 and 3, some participants reported that the one-hour session was too short. Participants were generally satisfied with the frequency of sessions. Overall, the participants appreciated the possibility of switching to a remote format because of the COVID-19 pandemic. Eight participants gave positive feedback about the treatment, identifying clear behavioral gains despite only partial or no symptomatic relief; S09, however, did not report positive feedback, behavioral gains or symptomatic relief. The relevance of the BATD intervention for everyday life was emphasized unanimously, whereas ATT was judged to have less transferability to everyday life, leading to a reduction in motivation to perform the exercises. Some barriers to engagement were reported by the participants, including the lockdown reducing their activities and social contacts. All participants reported being satisfied with the relationship with the therapist and with the therapist’s skills.

### Multiple measurement analysis

**Figure 3:**
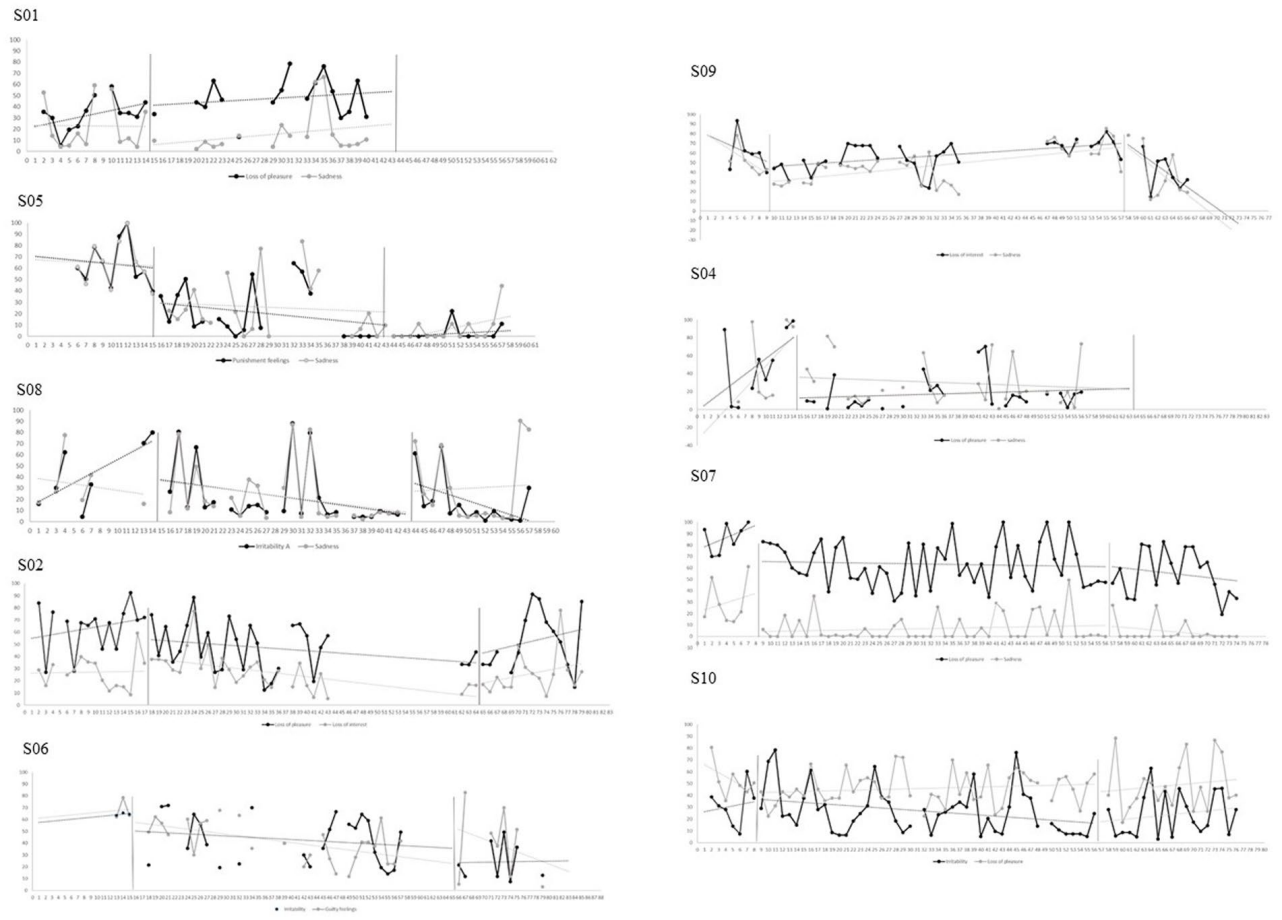
Raw data and trend for participants’ depressive symptoms ratings.

**Figure 4:**
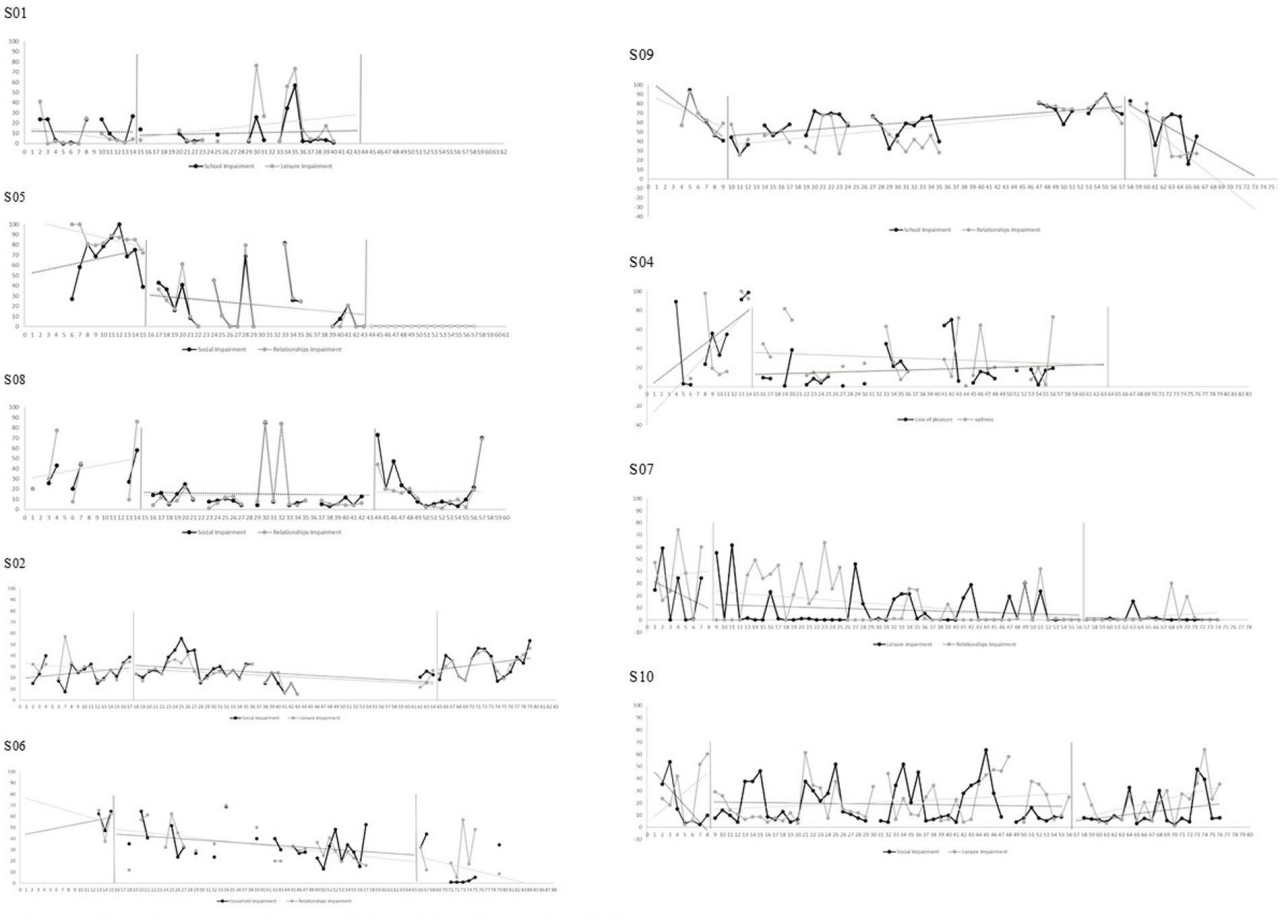
Raw data and trend for participants’ functioning impairment ratings.

**Figure 5:**
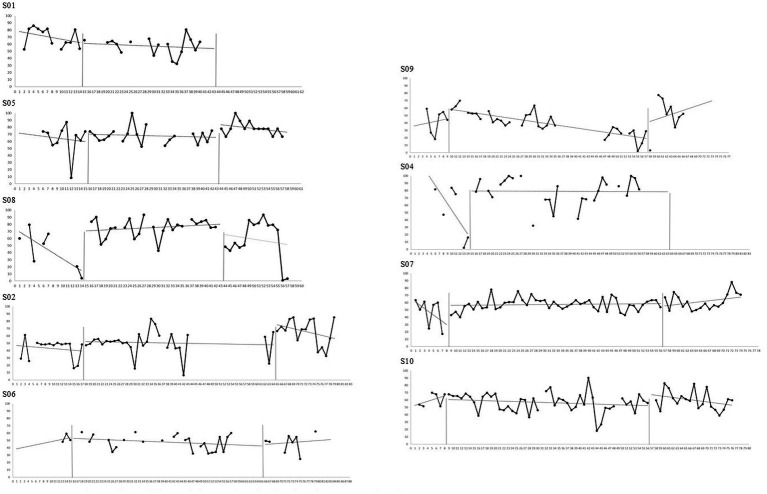
Raw data and trend for participants’ functioning impairment mood rating.

The means, standard deviations, Tau-U non-overlap indices, and Cohen’s d scores across phases are reported in Tables 4, 5. Graphs representing multiple measurement and associated trends are reported in Figures 3–5 for generic mental health measures and in Supplementary Material Section S2 for intervention’s-specific measures.

**Table 4:** Means, standard deviations, Tau-U and Cohen’s *d* scores for generic mental health measures for all participants across phases.

	Condition 1			Condition 2			Condition 3		
	S01	S05	S08	S02	S06	S09	S04	S07	S10
*Depressive symptoms 1*	*Loss of pleasure*	*Punishment feelings*	*Irritability*	*Loss of pleasure*	*Irritability*	*Loss of interest*	*Loss of pleasure*	*Loss of pleasure*	*Irritability*
Mean A (SD)	33.51 (13.97)	63.55 (19.69)	42.39 (28.80)	63.87 (18.89)	64.52 (1.08)	59.68 (19.12)	50.30 (37.34)	86.64 (12.74)	31.03 (17.35)
Mean B (SD)	48.01 (17.28)	20.38 (22.33)	22.04 (26.67)	47.33 (19.27)	43.17 (19.47)	57.18 (14.44)	17.52 (18.29)	63.33 (19.41)	26.72 (19.40)
Mean B′ (SD)	NA	2.38 (6.43)	17.43 (21.51)	59.46 (25.10)	24.19 (16.14)	44.53 (21.80)	NA	55.00 (19.82)	23.83 (18.56)
A-B Cohen’s d	1.04	−2.19	−0.71	−0.88	−19.85	−0.13	−0.88	−1.83	−0.25
A-B Tau-U	0.49^*^+	−0.83^***^	−0.52^*^	−0.51^***^	−0.60	0.04	−0.51^*^	−0.63^*^	−0.22
B-B′ Cohen’s d	NA	−0.81	−0.17	0.63	−0.97	−0.88	NA	−0.43	−0.15
B-B′ Tau-U	NA	−0.36 (c)	−0.17	0.30	−0.57^*^	−0.36	NA	−0.23	−0.09
*Depressive symptoms 2*	*Sadness*	*Sadness*	*Sadness*	*Loss of interest*	*Guilty feelings*	*Sadness*	*Sadness*	*Sadness*	*Loss of pleasure*
Mean A (SD)	22.85 (21.68)	63.87 (19.85)	40.41 (28.93)	27.17 (13.15)	68.10 (9.02)	51.43 (14.38)	49.62 (44.29)	29.49 (19.29)	52.53 (14.28)
Mean B (SD)	16.00 (19.02)	25.54 (25.78)	22.49 (26.30)	27.63 (15.14)	41.34 (16.49)	47.29 (17.99)	30.07 (24.58)	6.94 (11.63)	45.98 (13.45)
Mean B′ (SD)	NA	6.35 (12.10)	29.95 (33.18)	26.17 (17.51)	38.84 (29.93)	39.10 (27.29)	NA	3.98 (9.01)	48.48 (21.62)
A-B Cohen’s d	−0.32	−1.93	−0.62	0.04	−2.96	−0.29	−0.44	−1.17	−0.46
A-B Tau-U	−0.15	−0.74^***^	−0.51^*^	0.01	−0.90^*^	−0.12	−0.22	−0.74^***^	−0.23
B-B′ Cohen’s d	NA	−0.74	0.28	−0.10	−1.37	−0.46	NA	−0.26	0.19
B-B′ Tau-U	NA	−0.56^*^	0.03	−0.15	−0.06	−0.26	NA	−0.17	−0.03
*Funct. impair. 1*	*School impair.*	*Social impair.*	*Social impair.*	*Social impair.*	*Household* *impair.*	*School impair.*	*Household* *impair.*	*Leisure impair.*	*Social impair.*
Mean A (SD)	11.65 (11.48)	68.28 (22.01)	34.14 (14.42)	25.02 (9.27)	58.06 (9.37)	61.65 (19.22)	92.47 (9.44)	21.97 (22.73)	17.82 (19.54)
Mean B (SD)	10.44 (15.18)	21.51 (24.35)	15.32 (21.84)	26.00 (11.49)	34.74 (14.21)	60.53 (15.12)	70.90 (31.97)	8.21 (15.11)	18.75 (16.06)
Mean B′ (SD)	NA	0.00 (0.00)	22.58 (23.80)	32.69 (11.80)	15.19 (18.36)	56.26 (22.00)	NA	1.14 (3.61)	12.65 (13.63)
A-B Cohen’s d	−0.11	−2.13	−1.31	0.11	−2.48	−0.06	−2.29	−0.61	0.05
A-B Tau-U	0.00	−0.81^***^	−0.81^***^	0.00	−0.76^*^	0.04	−0.55^*^	−0.41	0.13
B-B′ Cohen’s d	NA	−0.88	0.33	0.58	−1.37	−0.28	NA	−0.47	−0.38
B-B′ Tau-U	NA	−0.65^*^	0.19	0.29	−0.53^*^	−0.09	NA	−0.25	−0.33^*^
*Funct. impair. 2*	*Leisure impair.*	*Relationship impair.*	*Familial impair.*	*Leisure impair.*	*Relationship impair.*	*Leisure impair.*	*Relationship impair.*	*Relationship impair.*	*Leisure impair.*
Mean A (SD)	7.62 (12.55)	86.02 (8.75)	39.46 (31.58)	28.96 (9.74)	55.20 (15.30)	64.70 (15.47)	74.19 (32.51)	37.17 (25.87)	29.03 (22.64)
Mean B (SD)	17.96 (25.34)	21.99 (26.44)	14.16 (22.18)	23.36 (8.52)	34.27 (15.55)	54.56 (19.11)	23.45 (23.06)	12.15 (18.08)	20.97 (15.93)
Mean B′ (SD)	NA	0.00 (0.00)	17.43 (18.63)	32.69 (9.29)	24.87 (19.11)	41.35 (2.07)	NA	2.96 (8.16)	20.77 (15.75)
A-B Cohen’s d	0.82	−7.32	−0.80	−0.57	−1.36	−0.66	−1.56	−0.97	−0.36
A-B Tau-U	0.38	−0.96^***^	−0.67^*^	−0.33	−0.75^*^	−0.29	−0.84^***^	−0.57^*^	−0.15
B-B′ Cohen’s d	NA	−0.83	0.33	1.10^+^	−0.60	−0.69	NA	−0.51	−0.01
B-B′ Tau-U	NA	−0.60^*^	0.19	0.52^*+^	−0.40	−0.37	NA	−0.30	−0.05
*Mood*									
Mean A (SD)	69.53 (13.11)	63.44 (21.47)	44.52 (27.47)	42.80 (13.3)	52.69 (5.69)	42.47 (16.40)	51.08 (35.30)	47.77 (18.89)	60.39 (8.90)
Mean B (SD)	57.37 (12.34)	68.12 (11.01)	75.54 (12.42)	50.66 (15.81)	47.78 (9.98)	40.04 (15.28)	78.89 (18.46)	57.63 (8.19)	56.57 (12.91)
Mean B′ (SD)	NA	78.57 (9.21)	58.45 (28.96)	65.93 (17.48)	47.04 (12.35)	50.12 (23.57)	NA	61.65 (11.01)	59.70 (12.71)
A-B Cohen’s d	−0.93	0.22	1.13	0.59	0.86	−0.15	0.79	0.52	−0.43
A-B Tau-U	−0.44^*+^	−0.05	0.70^*^	0.36^*^	−0.21	−0.12	0.48	0.24	−0.22
B-B′ Cohen’s d	NA	0.95	−1.38	0.97	−0.07	0.66	NA	0.49	0.24
B-B′ Tau-U	NA	0.61^*^	−0.33	0.51^*^	−0.03	0.35	NA	0.17	0.06

Funct. impair., Functioning impairment; d, Cohen’s d score; Tau, Tau for non-overlap with baseline trend control; +, significant deterioration, NA, missing data; (c), corrected for baseline trend;

* *p* < 0.05;

*** *p* < 0.001.

**Table 5:** Means, standard deviations, Tau-U and Cohen’s *d* scores for intervention’s-specific measures for all participants across phases.

	Condition 1			Condition 2			Condition 3		
	S01	S05	S08	S02	S06	S09	S04	S07	S10
*Behavioral Activation*									
Mean A (SD)	78.49 (12.94)	70.43 (16.34)	56.11 (23.15)	37.85 (14.88)	54.12 (9.39)	28.85 (17.99)	68.70 (32.15)	36.56 (25.77)	31.80 (17.73)
Mean B (SD)	70.52 (8.11)	48.60 (15.17)	70.21 (15.66)	50.98 (16.57)	53.09 (11.19)	37.02 (13.51)	89.35 (10.95)	34.86 (25.63)	43.10 (15.15)
Mean B′ (SD)	NA	69.05 (7.77)	72.43 (21.82)	58.57 (16.96)	45.43 (3.89)	38.72 (14.14)	NA	45.56 (22.98)	45.25 (16.83)
A-B d	−0.62	−1.34	0.61	0.88	−0.11	0.45	0.64	−0.07	0.64
A-B Tau-U	−0.50^*+^	−0.73^***+^	0.44	0.40^*^	−0.09	0.26	0.48^*^	−0.06	0.35
B-B′ d	NA	1.35	0.4	0.46	−0.68	0.13	NA	0.42	0.14
B-B′ Tau-U	NA	0.98^***^(c)	0.17	0.25	−0.53^*+^	0.06	NA	0.23	0.08
*Behavioral Avoidance*									
Mean A (SD)	19.09 (15.02)	63.23 (7.43)	36.05 (29.28)	29.96 (8.64)	34.41 (2.15)	43.37 (25.62)	24.61 (30.77)	3.38 (5.43)	35.64 (18.08)
Mean B (SD)	13.98 (5.24)	51.51 (13.89)	15.50 (14.25)	41.25 (17.54)	26.97 (14.78)	56.68 (12.30)	13.23 (13.74)	20.81 (25.88)	25.95 (17.88)
Mean B′ (SD)	NA	58.73 (9.17)	5.76 (3.74)	39.21 (19.95)	3.23 (4.06)	51.23 (10.78)	NA	0.70 (0.90)	16.07 (11.14)
A-B d	−0.34	−1.58	−0.70	1.31	−3.46	0.52	−0.37	3.21	−0.54
A-B Tau-U	−0.11	−0.54^*^	−0.58^*^	0.23 (c)	−0.36	0.31	−0.07	0.56^+^(c)^*^	−0.33
B-B′ d	NA	0.52	−0.68	−0.12	−1.61	−0.44	NA	−0.78	−0.55
B-B′ Tau-U	NA	0.30	−0.61^***^	0.18	−0.97^***^	−0.32	NA	−0.54^***^	−0.37
*Self-focused attention*									
Mean A (SD)	39.61 (19.27)	61.29 (10.49)	15.80 (6.66)	29.61 (13.12)	46.95 (9.70)	38.53 (13.96)	71.21 (19.83)	64.06 (22.07)	30.88 (10.66)
Mean B (SD)	52.06 (10.63)	45.48 (17.52)	46.06 (24.50)	45.07 (18.79)	37.71 (17.73)	40.08 (15.40)	76.97 (25.74)	69.97 (11.75)	38.43 (17.13)
Mean B′ (SD)	NA	48.41 (10.32)	62.06 (27.58)	35.61 (18.91)	13.44 (9.89)	36.62 (21.73)	NA	69.03 (8.67)	30.35 (19.01)
A-B Cohen’s d	0.65	−1.51	4.54	1.18	−0.95	0.11	0.29	0.27	0.71
A-B Tau-U	0.41	−0.58^*^	0.81^*^^+^	0.49^*^^+^	−0.32	−0.04	0.28	0.20	0.29
B-B′ Cohen’s d	NA	0.17	0.65	−0.50	−1.37	−0.22	NA	−0.08	−0.47
B-B′ Tau-U	NA	0.12	0.35	−0.32	−0.82^***^	−0.28	NA	−0.04	−0.25
*Rumination*									
Mean A (SD)	19.35 (15.63)	62.04 (25.15)	46.41 (22.62)	32.54 (12.52)	70.97 (6.72)	38.71 (14.81)	44.92 (40.74)	35.33 (30.61)	15.82 (12.03)
Mean B (SD)	21.88 (17.86)	30.11 (21.12)	19.13 (13.71)	39.93 (18.31)	36.01 (18.67)	48.20 (14.32)	42.94 (25.93)	7.91 (10.85)	28.75 (20.39)
Mean B′ (SD)	NA	11.90 (14.10)	34.72 (37.00)	37.68 (15.96)	21.37 (19.97)	35.09 (25.66)	NA	2.01 (5.20)	28.46 (19.16)
A-B Cohen’s d	0.16	−1.27	−1.21	0.59	−5.21	0.64	−0.05	−0.90	1.07
A-B Tau-U	0.12	−0.69^*^	−0.74^*^	0.23	−0.91^*^	0.33	0.06	−0.79^***^	0.44
B-B′ Cohen’s d	NA	−0.86	1.14	−0.15	−0.78	−0.92	NA	−0.54	−0.01
B-B′ Tau-U	NA	−0.55^*^	0.02	0.25	−0.46	−0.42	NA	−0.38^*^	−0.03

d = Cohen’s d score; Tau = Tau for non-overlap with baseline trend control; + = significant deterioration, NA = missing data; (c) = corrected for baseline trend;

* *p* < 0.05;

*** *p* < 0.001.

Visual and statistical analyses suggest that, relative to baseline, six out of nine participants made significant improvements in at least one personal depressive symptom; five improved at least one personal life functioning aspect; and two demonstrated a positive change in their general mood over the course of treatment (S02 and S08). However, generic mental health measures appear unchanged for S01 (Condition 1) and S10 (Condition 3) and appear to be worsening for S09 (Condition 2). By considering the two-week follow-up period, additional significant improvements are reported in both symptoms, both functioning aspects and mood for S05 (Condition 1); in mood for S02 (Condition 2); and in one symptom for S06 (Condition 2). The three Conditions seemed to have similar results on generic mental health measures.

For intervention’s-specific measures, visual and statistical analyses suggest that, relative to baseline, two out of nine participants experienced significant increases in behavioral activation (S02 and S04); two significant reductions in behavioral avoidance (S05 and S08); one a reduction in self-focused attention (S05); and four reductions in rumination. However, S01 and S05 (Condition 1) reported a deterioration of behavioral activation; S07 (Condition 3) an increase in behavioral avoidance; and S08 and S02 (Condition 1 and 2, respectively) an increase in self-focused attention. Again, intervention-specific measures appear unchanged for S01 (Condition 1) and S10 (Condition 3) and appear to be worsening for S09 (Condition 2). By considering the two-week follow-up period, one additional significant increase in behavioral activation was reported (S05); three significant reductions of behavioral avoidance (S08, S06 and S07); one reduction in self-focused attention (S06); and two reductions of rumination (S05 and S07). The three Conditions seemed to have similar results on intervention’s-specific measures.

### Component analyses of each treatment

To control for the specificity of treatments, we analyzed ATT and BATD separately in sequential conditions. The means, standard deviations, Tau-U non-overlap indices, and Cohen’s *d* scores for intervention’s-specific measures across treatment components in Conditions 2 and 3 are reported in the Supplementary materials (Section 3 and Section 5). Graphs representing multiple measurement and associated trends are reported in Section 4 and Section 6.

As expected in Condition 2, S06 first reported a reduction in rumination during the ATT. However, S02 reported an unexpected increase in self-focused attention, rumination, behavioral activation and behavioral avoidance during the ATT relative to baseline. With the introduction of BATD, S02 and S06 reported a reduction in rumination but S06 also reported a significant reduction in self-focused attention. S09 reported no significant change during both phases. As expected in Condition 3, S04 reported a significant increase in behavioral activation, and S07 reported a reduction in rumination during BATD. However, S07 also reported an unexpected increase in behavioral avoidance. The ATT did not provide any additional improvements over the BATD. S10 reported no significant change during both phases.

To compare the effectiveness of BATD in the three different formats, we compared the generic mental health measures results associated with A-B phases in Condition 1, and A-BATD phases in Conditions 2 and 3. The effectiveness of BATD did not seem to differ according to condition.

### Analyses of pre–post-measurements

We used standardized questionnaires to measure generic mental health measures (depression, work impairment, social impairment, and well-being) and intervention-specific measures (behavioral activation, behavioral avoidance, rumination, behavioral measures of attentional control and self-reported measures of attentional control).

Participants’ total scores on the standardized measures administered at each phase and the number of participants with reliable changes are reported in Table 6.

**Table 6:** Participants’ total scores on standardized measures administered at each phase and number of participants with reliable changes.

		Condition 1				Condition 2				Condition 3				Total	
Measures		S01	S05	S08	RC I n	S02	S06	S09	RC I n	S04	S07	S10	RC I n	RC I n	RC D n
BDI-II RCI (7.63)	Pre	13	26	25	–	18	25	33	–	22	13	11	–	*–*	*–*
	Post	2^*^	8^*^	11^*^	3/3	6^*^	2^*^	35	2/3	15	5^*^	7	1/3	6/9	0/9
	2 weeks	2^*^	8^*^	11^*^	3/3	8^*^	1^*^	22^*^	3/3	11^*^	4^*^	6	2/3	8/9	0/9
	3 months	7	31	11^*^	1/3	10^*^	26	7^*^	2/3	12^*^	5^*^	6	2/3	5/9	0/9
BADS-Work imp. RCI (8.04)	Pre	2	11	0	–	4	19	24	–	17	6	15	–	*–*	*–*
	Post	2	10	2	0/3	4	9^*^	23	1/3	9	3	9	0/3	1/9	0/9
	2 weeks	0	3	2	0/3	3	5^*^	22	1/3	12	16^+^	14	0/3	1/9	1/9
	3 months	10	19	2	0/3	7	20	19	0/3	11	6	13	0/3	0/9	0/9
BADS- Social imp. RCI (6.53)	Pre	2	29	8	–	8	19	10	–	4	22	3	–	*–*	–
	Post	4	1^*^	14	1/3	3	8^*^	5	1/3	4	4^*^	3	1/3	3/9	0/9
	2 weeks	4	5^*^	23^+^	1/3	9	5^*^	4	1/3	4	9^*^	1	1/3	3/9	1/9
	3 months	1	4^*^	10	1/3	5	22	3^*^	1/3	6	8^*^	0	1/3	3/9	0/9
WEMWBS RCI (7.02)	Pre	40	36	42	–	39	38	36	–	40	43	41	–	–	–
	Post	48^*^	49^*^	42	2/3	48^*^	52^*^	36	2/3	44	52^*^	42	1/3	5/9	0/9
	2 weeks	51^*^	49^*^	39	2/3	48^*^	53^*^	37	2/3	50^*^	45	41	1/3	5/9	0/9
	3 months	37	28^+^	47	0/3	40	34	47^*^	1/3	48^*^	50	39	1/3	2/9	1/9
AERT RCI (3.84)	Pre	17	20	18	–	15	19	16	–	15	16	12	–	–	–
	Post	13^*^	14^*^	14^*^	3/3	17	14^*^	19	1/3	16	13	13	0/3	4/9	0/9
	2 weeks	15	15^*^	16	1/3	8^*^	17	20^+^	1/3	11^*^	12^*^	7^*^	3/3	5/9	1/9
	3 months	15	17	15	0/3	12	18	21^+^	0/3	14	6^*^	11	1/3	1/9	1/9
BADS-Behav. Activation RCI (10.00)	Pre	28	28	22	–	13	14	14	–	27	26	13	–	–	–
	Post	33	26	17	0/3	27^*^	33^*^	20	2/3	34	29	13	0/3	2/9	0/9
	2 weeks	37	28	20	0/3	23^*^	31^*^	15	2/3	38^*^	18	8	1/3	3/9	0/9
	3 months	18^+^	11^+^	12^+^	0/3	15	14	24^*^	1/3	38^*^	29	11	1/3	2/9	3/9
BADS- Behav. Avoidance RCI (7.42)	Pre	10	14	9	–	6	12	12	–	18	8	12	–	–	–
	Post	1^*^	6^*^	16	2/3	0	6	17	0/3	8^*^	6	10	1/3	3/9	0/9
	2 weeks	1^*^	7	8	1/3	1	8	16	0/3	7^*^	1	2^*^	2/3	3/9	0/9
	3 months	4	4^*^	3	1/3	3	21^+^	12	0/3	7^*^	6	7	1/3	2/9	1/9
PASAT RCI (8.25)	Pre	42	50	54	–	42	53	55	–	56	53	40	–	–	–
	Post	46	47	55	0/3	60^*^	59	57	1/3	56	59	48	0/3	1/9	0/9
	2 weeks	48	43	58	0/3	59^*^	59	56	1/3	58	60	54^*^	1/3	2/9	0/9
	3 months	50	53	55	0/3	59^*^	58	55	1/3	58	59	44	0/3	1/9	0/9
ASQ- IAS RCI (7.60)	Pre	26	23	25	–	27	34	35	–	32	20	29	–	–	–
	Post	23	25	14^*^	1/3	26	30	34	0/3	29	15	35	0/3	1/9	0/9
	2 weeks	26	23	12^*^	1/3	25	28	33	0/3	28	15	27	0/3	1/9	0/9
	3 months	30	26	19	0/3	27	32	39	0/3	30	17	25	0/3	0/9	0/9
ASQ-EAS RCI (6.56)	Pre	11	27	22	–	12	18	17	–	26	22	22	–	–	–
	Post	17	26	20	0/3	17	13	17	0/3	25	16	19	0/3	0/9	0/9
	2 weeks	17	24	18	0/3	14	17	13	0/3	19^*^	20	18	1/3	1/9	0/9
	3 months	18^+^	24	21	0/3	20^+^	19	18	0/3	24	22	18	0/3	0/9	2/9

BDI-II, Beck Depression Inventory – Second Edition; BADS-Work imp., Behavioral Activation for Depression Scale-Work/School Impairment subscale; BADS-Social imp., Behavioral Activation for Depression Scale-Social impairment subscale; WEMWBS, Warwick-Edinburgh Mental Well-Being Scale; AERT, Abstract Evaluative mode of Repetitive Thinking subscale; FU, follow-up assessment; * = reliable improvement (relative to pre-assessment); ^+^ = reliable deterioration (relative to pre-assessment); RCI, Reliable Change Improvement; RCD, Reliable Change Deterioration. BADS-Behav. Activation, Behavioral Activation for Depression Scale- Behavioral Activation subscale; BADS-Behav. Avoidance, Behavioral Activation for Depression Scale-Behavioral Avoidance subscale; PASAT, Paced Auditory Serial-Addition Task; ASQ-IAS, Attentional Style Questionnaire-Internally oriented Attention Subscale; ASQ-EAS, Attentional Style Questionnaire-Externally oriented Attention Subscale.

For generic mental health measures, pre–post-measurements suggest that six participants reported a reliable decrease in depressive symptoms. Two weeks after the intervention, two additional participants reported a reliable decrease in depressive symptoms, for a total of three in Conditions 1 and 2 and two in Condition 3. One participant reported a reliable decrease in work impairment (S06 in Condition 2); three reported a reliable decrease in social impairment (one in each condition); and five participants reported a reliable increase in well-being. Two weeks after the intervention, one additional participant reported a reliable increase in well-being (S04), for a total of two improvements in Condition 1 and 2 and one in Condition 3. S09 (Condition 1), S04 and S10 (Condition 3) did not report any reliable change immediately after the intervention. Every significant change in generic mental health measures reported immediately after the intervention was present 2 weeks later except for one score of well-being (S07). However, only a minority of changes were still present 3 months later. Conditions 1 and 2 seemed to have better results than Condition 3 on generic mental health measures.

For intervention’s-specific measures, pre–post-measurements suggest that four participants reported a reliable decrease in rumination (three in Conditions 1 and one in Condition 2). Two weeks after the intervention, four additional participants reported a reliable decrease in rumination (one in Condition 2 and three in Condition 3). Two participants reported a reliable increase in behavioral activation (S02 and S06 in Condition 2); three a reliable decrease in behavioral avoidance (S01 and S05 in Condition 1 and S04 in Condition 2); one reported a reliable increase in attentional control (S02 in Condition 2) and one a decrease in internal attentional style (S08 in Condition 1). The three changes of behavioral activation, two changes of behavioral avoidance (S01 and S04), and attentional control and internal attentional style changes were maintained 2 weeks later. Only rumination for S05 and behavioral avoidance for S04 were still present 3 months later.

Conditions 1 and 2 seemed to have better results than Condition 3 on generic mental health measures. Furthermore, Condition 1 seems to be associated with a higher rumination response rate immediately after the intervention.

### Inter-individual differences in response to treatment

Of the nine participants, only one did not respond at all to the treatment (S10), one participant respond 2 weeks later (S04) and one participant respond 3 months later (S09). These three participants did not seem to share any common demographic or clinical characteristics. S04 and S09’s failure to do homework might explain their non-response to treatment immediately after the intervention. However, S10 reported a high level of homework compliance. S10 reported relatively low levels of symptoms, impairments and disturbance in psychological processes before the intervention. Based on qualitative inspection, individual factors such as age, gender, clinical status before treatment, history of depression, sociodemographic status, education level, pre-treatment level of attentional control, self-focused attention, rumination, behavioral activation, behavioral avoidance did not seem to be related to the response rates.

## Discussion

The first objective of this study was to investigate whether BATD combine to ATT can be efficient in order to reduce depressive symptoms and improve well-being and life functioning in the short (2 weeks) and longer term (3 months), as measured by generic mental health measures (depressive symptoms, well-being and life functioning) and intervention’s-specific measures (behavioral activation, behavioral avoidance, self-focused attention, attentional control and rumination). The second objective was to investigate which combination of treatment produced the best outcomes (ATT and BATD concurrently, ATT followed by BATD, or BATD followed by ATT).

This study was associated with no dropouts, acceptable level of treatment attendance and homework compliance and satisfaction with the intervention. Treatment attendance and homework compliance were higher in Conditions 1 and 3, where the treatment started with BATD, which is consistent with previous empirical data reporting high levels of adherence and acceptability for participants involved in BATD (McIndoo et al., 2016; Simmonds-Buckley et al., 2019). The ATT adherence was also acceptable, which is encouraging because cognitive training adherence had sometimes been identified as a challenge in previous studies (Vervaeke et al., 2018).

For generic mental health measures, visual and statistical analyses showed that, relative to baseline, six out of nine participants made significant improvements in at least one personal depressive symptom, five improved at least one personal life functioning aspect, and two demonstrated a positive change in their general mood over the course of treatment. Three participants did not respond to the treatment during the intervention (S01, S09 and S10). Regarding pre–post-measurements, six out of nine participants responded to treatment for depression symptoms; one responded to treatment for work impairment; three responded to treatment for social impairment; and five responded for well-being. Two weeks after the intervention, two additional participants responded to treatment for depressive symptoms and one for well-being. Every significant change in generic mental health measures reported after the intervention was present 2 weeks later except for one score of well-being. However, only a minority of changes were still present 3 months later. Conditions 1 and 2 seemed to have better results than Condition 3 on generic mental health measures.

The exploration of inter-individual differences suggest that homework completion did not seem to be related to the response to treatment. Furthermore, S10 who did not respond to treatment reported relatively low levels of symptoms, impairments and disturbance in psychological processes before the intervention. A floor effect might then explain the lack of improvement in these measures. Gender, clinical status before treatment, history of depression, sociodemographic status, education level, pre-treatment level of attentional control, self-focused attention, rumination, behavioral activation, behavioral avoidance did not seem to be related to the response rates that is consistent with recent findings reported in the BATD literature (Ekers et al., 2014; Cuijpers, 2017; Simmonds-Buckley et al., 2019).

For intervention’s-specific measures, visual and statistical analyses showed that, relative to baseline, four of the nine participants experienced improvements in rumination, three in behavioral activation, two in behavioral avoidance, and two in self-focused attention. According to pre–post-measurements, four of the nine participants were considered treatment responders for rumination; two responded to treatment for behavioral activation; three for behavioral avoidance, and one for attentional control and internal attentional style, but none for external attentional style. Three participants also reported a reduction in rumination 2 weeks after the end of the intervention. Of the nine participants, only S09 did not report any positive change in rumination. It is interesting that responses sometimes differed in the multiple measurements and pre–post-evaluations. The nature of the evaluations might explain this divergence, with a higher level of content validity for multi-item scales than for one item. The two measures might assess different facets of the construct. According to the multiple measurements, almost every significant change in rumination reported during the intervention was present during the follow-up period 2 weeks later. However, only a minority of changes were still present 3 months later. None of the conditions seemed to produce better outcomes than the others, with the exception of Condition 1 where rumination was associated with a better response to treatment immediately after the intervention. If the measures in the follow-up phase had been considered, all conditions would have been associated with similar response rates. Together, these results suggest that all three formats may improve rumination for most participants, but the concurrent format may have a greater effect immediately after the intervention than the two sequential formats. The more compact nature of the first format may have elicited more cognitive resources during the sessions, which may have boosted the effect of the intervention in comparison with the other conditions. Furthermore, the positive reinforcements and hedonic experiences could have increased motivation, leading to enhanced attentional resources, as suggested by previous studies showing a relationship between motivation and cognitive performance (for a review, see Botvinick and Braver, 2015).

Our results suggest that Conditions 1 and 3 seem to be better than Condition 2 in terms of adherence and that Condition 1 seems to be better than the others regarding rumination response rates immediately after the intervention. Overall, our findings suggest that five sessions of concurrent treatment could be a better option than sequential formats in order to reduce rumination immediately after the intervention, even though all three formats improved rumination for a majority of participants.

Our results do not support theoretical models related to BATD or ATT whereby BATD is said to target behavioral activation, and behavioral avoidance while ATT targets attentional control and self-focused attention. The component analysis of each treatment revealed that BATD was not associated with a consistent change in behavioral activation or behavioral avoidance across participants. Similarly, ATT was not associated with a consistent change of self-focused attention or attentional control across participants. Together, these findings do not support the specificity of these two treatments.

In this study, neither behavioral activation nor behavioral avoidance seemed to act as a clear mechanism of change although rumination could act as a mechanism of change for some participants (e.g., S01.S05, S08 S06 S04). However, other participants reported a reduction in depressive symptoms without a reduction in rumination (S09), reported a reduction in rumination that not precede temporally the reduction in depressive symptoms (S02 and S07) or reported a reduction in rumination without a significant change of depressive symptoms (S10).

Moreover, the inspection of response rates for generic mental health measures and intervention-specific measures in different BATD phases suggest that the effectiveness of BATD seems to be similar in each format and that none of the formats enhances its effectiveness. Even though unexpected, our findings are consistent with past studies that reported that cognitive training added to BATD or another usual treatment did not seem to potentiate the change in depressive symptoms or rumination in depressed samples (Moshier and Otto, 2017; Ferrari et al., 2021).

The study was conducted during the lockdown due to the second wave of COVID-19. The spread of the COVID-19 pandemic had a serious impact on people’s mental health; with a much higher mean prevalence of depression than before (31.4% or 33.7% in Europe) (Salari et al., 2020; Wu et al., 2021). The pandemic was associated with higher levels of depressive symptoms, stress, mood swings, irritability, and insomnia (Brooks et al., 2020). This context has decreased access to pleasant activities and social interactions that may have reduced access to important sources of positive reinforcement. This limitation may have reduced BATD’s influences on behavioral activation. Moreover, our intervention may have protected the participants against a deterioration in their mental health and well-being during the lockdown period. Indeed, it is possible that our results would have been different in a more favorable context than the health crisis and the lockdown.

Some limitations of this study should be considered. First, the self-report measurement of outcomes might be subject to retrospective recall biases (Rinner et al., 2019) that could have distorted the subjective perception of our variables. Furthermore, participants were not randomly assigned to one baseline duration and the baseline was not controlled for stability. Future studies may benefit from using a random assignment of participants to baseline duration and eventually a response-guided design to ensure a stable baseline (Joo et al., 2018). Moreover, 10 sessions of ATT may not have activated prefrontal regions to the extent necessary to affect mood and symptoms. The optimal dosage of ATT remains unknown (Fergus and Bardeen, 2016). Another limitation is that this study included only a pre–post-measurement of attentional control resources, given the difficulty of measuring this factor daily. However, this process may have acted as a mechanism of change even if pre–post-measurements did not report consistent attention control changes. Moreover, we followed the recommendation of a minimum of three participants per condition to demonstrate its effect but additional participants would have enhanced the validity of our finding. The number of participants also limited the exploration of individual factors that may influence the effectiveness of the intervention. Future studies may benefit from identifying moderators of the effect of the intervention at the individual level. Additionally, as suggested in the literature, the simple fact of being involved in a process of self-monitoring may have enhanced participants’ engagement in the therapeutic process that could influence symptoms and outcome improvement (van Os et al., 2017). In the same line, the present findings could have been influenced by the effect of time. Indeed, previous studies have reported that patients’ total depression scores tent to naturally decline over time after their first evaluation (Fried et al., 2016). Finally, as participants are self-referred people from the general population, and that only one male participated, future studies may benefit from exploring the generalizability of our findings with the inclusion of more clinical patients referred for treatment, more men, and participants with comorbid diagnoses, who are under-represented in our sample.

This study tested whether combining BATD with ATT is an efficient treatment up to 3 months later, documenting which combination of treatment produced the best outcome and investigating the mechanisms of action underlying the effectiveness of treatment. The design used allowed us to assess the specificity of treatment components and the potential mechanisms of change. Future studies should investigate how to enhance the long-term therapeutic effects (e.g., adding booster sessions) and continue to explore the mechanisms of change in relation to inter-individual differences with mediation analysis. Indeed, proof of the causal role of specific factors on outcomes is lacking, and there is insufficient evidence that these specific factors are core elements of how psychotherapy works and for whom. A promising research strategy to overcome these limitations would be the use of ambulatory assessment or ecological momentary assessment (Gloster et al., 2017), which make it possible to assess variations in behaviors within much smaller time frames and with less retrospective recall bias.

## Conclusion

Overall, our results demonstrate a feasible, acceptable combined BATD and ATT intervention with significant positive clinical short-term benefits in terms of depression symptoms, functioning, well-being, and rumination for a majority of adults with depressive symptoms with only one participant who did not respond to the treatment. Benefits were maintained 2 weeks after the intervention, but not 3 months later. Our data also suggest that five sessions of concurrent treatment could be a better option than sequential formats for treatment adherence, response to generic mental health measure and rumination immediately after the intervention. Furthermore, BATD did not appear to have a specific effect on behavioral activation, behavioral avoidance and rumination while ATT did not seem to have a specific effect on self-focused attention and rumination.

## Data availability statement

The original contributions presented in the study are included in the article/supplementary material, further inquiries can be directed to the corresponding author.

## Ethics statement

The studies involving human participants were reviewed and approved by The Ethics Committee of Université de Liège. The patients/participants provided their written informed consent to participate in this study.

## Author Contributions

AK: conceptualization, methodology, investigation, formal analysis, writing—original draft, visualization, and review and editing. MG: conceptualization, methodology, formal analysis, and writing—review and editing. EL: investigation. SB: conceptualization, methodology, supervision, project administration, and writing–review and editing. All authors contributed to the article and approved the submitted version.

## Conflict of interest

The authors declare that the research was conducted in the absence of any commercial or financial relationships that could be construed as a potential conflict of interest.

## Publisher’s note

All claims expressed in this article are solely those of the authors and do not necessarily represent those of their affiliated organizations, or those of the publisher, the editors and the reviewers. Any product that may be evaluated in this article, or claim that may be made by its manufacturer, is not guaranteed or endorsed by the publisher.

## Footnotes

^1^This assignment was respected except for S08 who was in quarantine during the first appointment and for whom the appointment was postponed to the following week.

^2^Registration number: NCT04595539; title: “Does Attention Training Technique Enhance the Efficacy of Behavioral Activation Treatment for Depression: A Multiple Baseline Study.”

^3^Approval number: 1920-119 (29/08/2020).

^4^The evaluator was intensively trained by the first and the last authors to administer the Mini-International Neuropsychiatric Interview (Lecrubier et al., 1997). The training included role-play sessions (at least 22 h sessions) and several supervised practice sessions before the start of the experiment.

^5^Philippot, P., Verschuren, A., and Douilliez, C. (2020). Trans-diagnostic processes in depression and anxiety: assessing differentiated cognitive modes in repetitive thinking. Manuscript submitted for publication.

^6^M.-N. Levaux, PhD in Psychology, email: mnlevaux@uliege.be.

^7^This adjustment was due to COVID-19 lockdown. Indeed, at the time, health care recommendations indicated that we could continue with face-to-face, but remote format was also recommended. So we left the choice to our participants concerning the intervention format.
