# Supplementary material for: Is the combination of behavioral activation and attention training technique effective to reduce depressive symptomatology? A multiple case study
Source: Front Psychol. 2022 Jul 20;13:914094. doi: 10.3389/fpsyg.2022.914094 (PMC9350628; doi:10.3389/fpsyg.2022.914094)

|  |
| --- |

| Table S1. Common measures collected daily | | | | |
| --- | --- | --- | --- | --- |
| ***Construct*** | **French item** | **English item** | **Extremity of VAS** |  |
| ***Behavioral Activation*** | J’ai été une personne active et j’ai accomplie les objectifs que je m’étais fixés | I was an active person and accomplished the goals I set out to do. | Not at all - Completely | Item 6 of the BADS |
| ***Behavioral Avoidance*** | La plupart des choses que j’ai faites avaient pour objectif d’échapper ou d’éviter quelque chose de désagréable | Most of what I did was to escape from or avoid something unpleasant. | Not at all - Completely | Item 8 of the BADS |
| ***Self-focused attention*** | J’ai focalisé mon attention sur moi-même plutôt que sur mon environnement externe | I focused my attention on myself rather than on my external environment | Not at all - Completely | - |
| ***Rumination*** | J’ai passé du temps à ressasser mes problèmes | I spent a long time thinking over and over about my problems. | Not at all - Completely | Item 6 of the BADS |
| ***Mood*** | Mon humeur générale au cours de cette journée a été | My general mood during this day was | Very negative – Very positive | - |
| *Note*. VAS = Visual Analogue Scale ; BADS = Behavioral Activation for Depression Scale | | | | |

S2. (1) Raw data and trend for participants' behavioral activation ratings.

S01


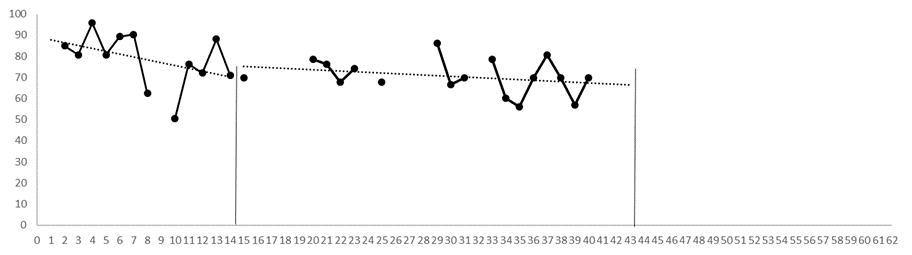


S05


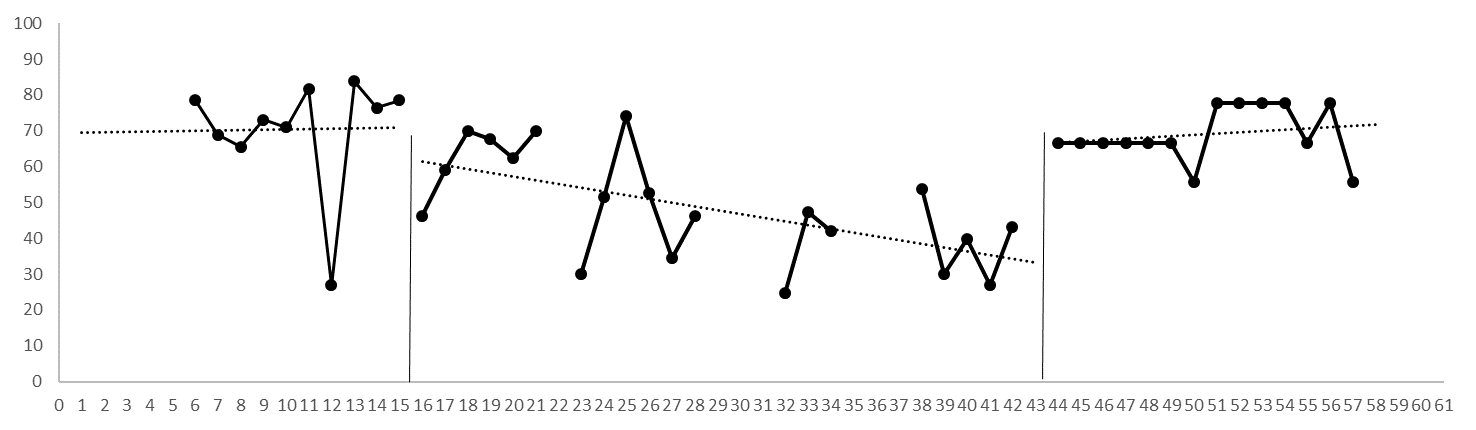


S08


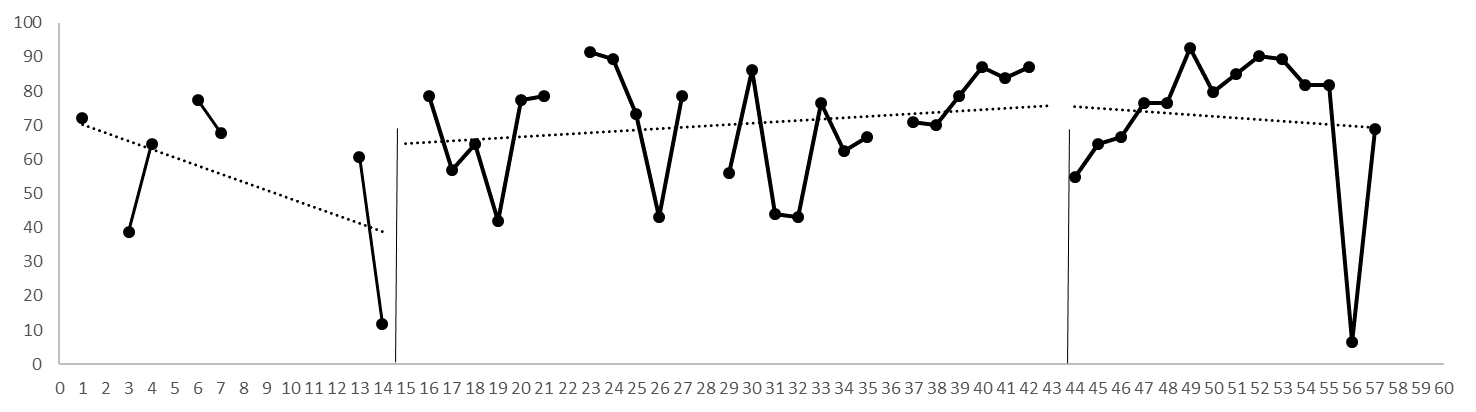


S02


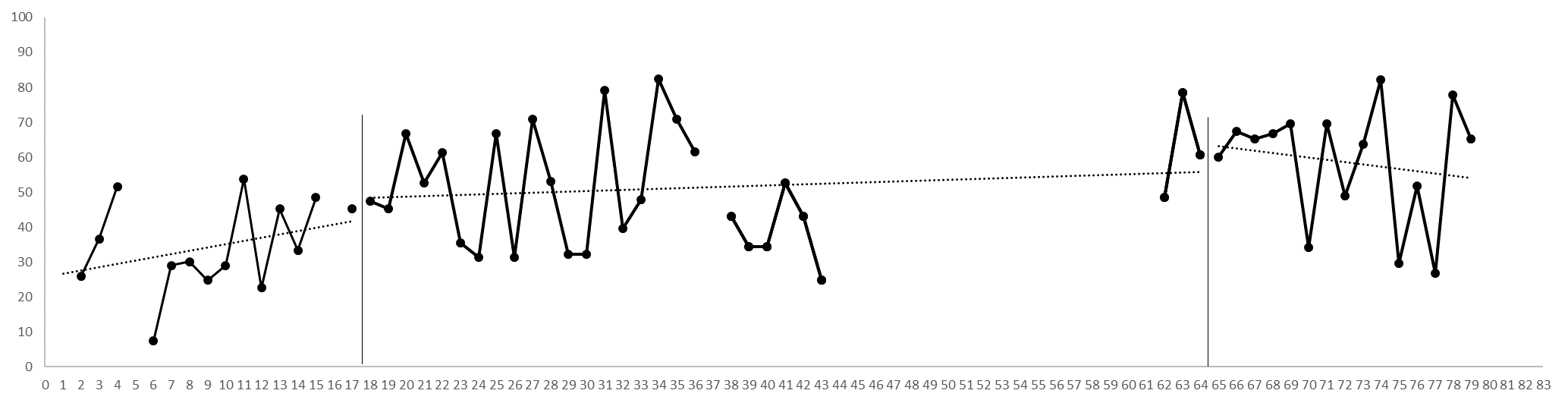


S06


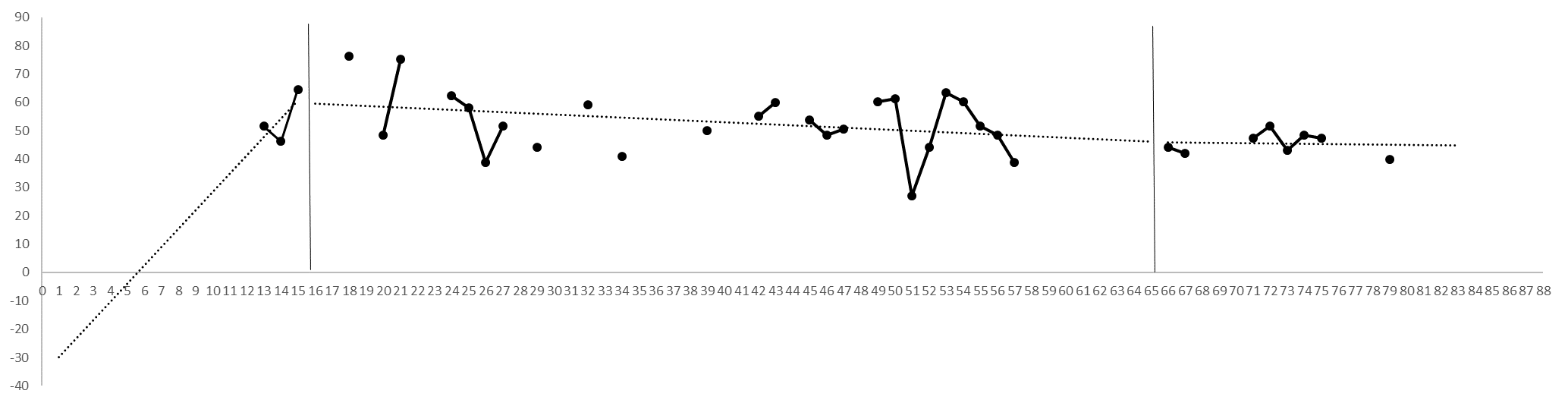


S09


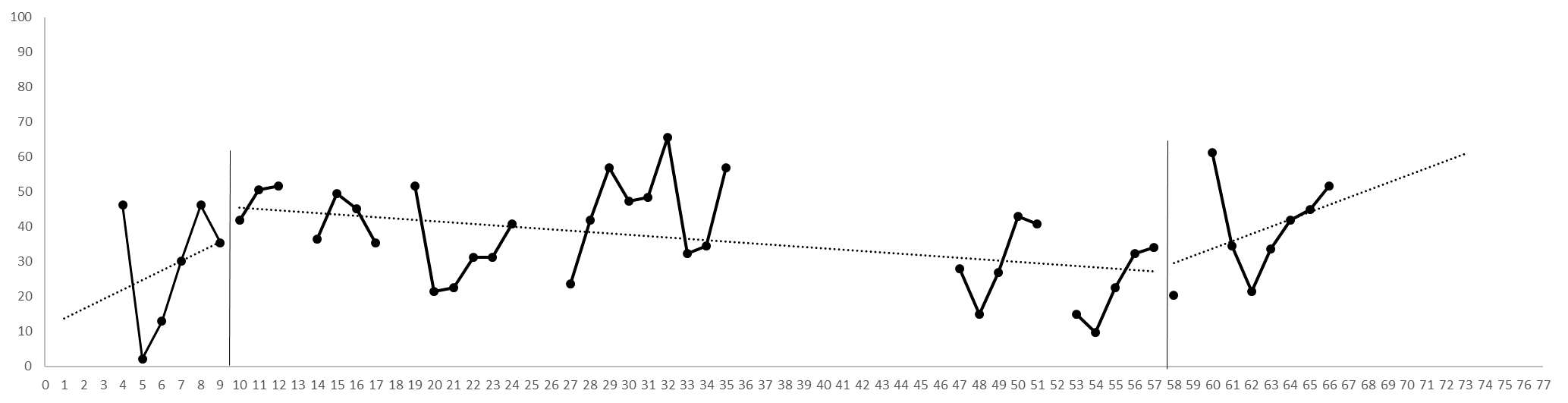


S04


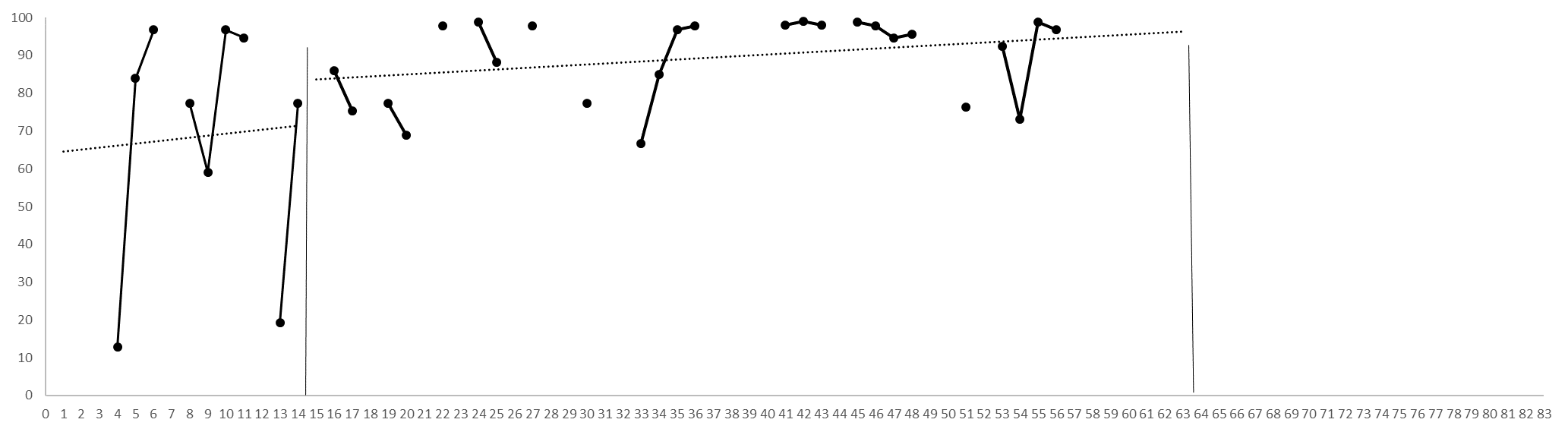


S07


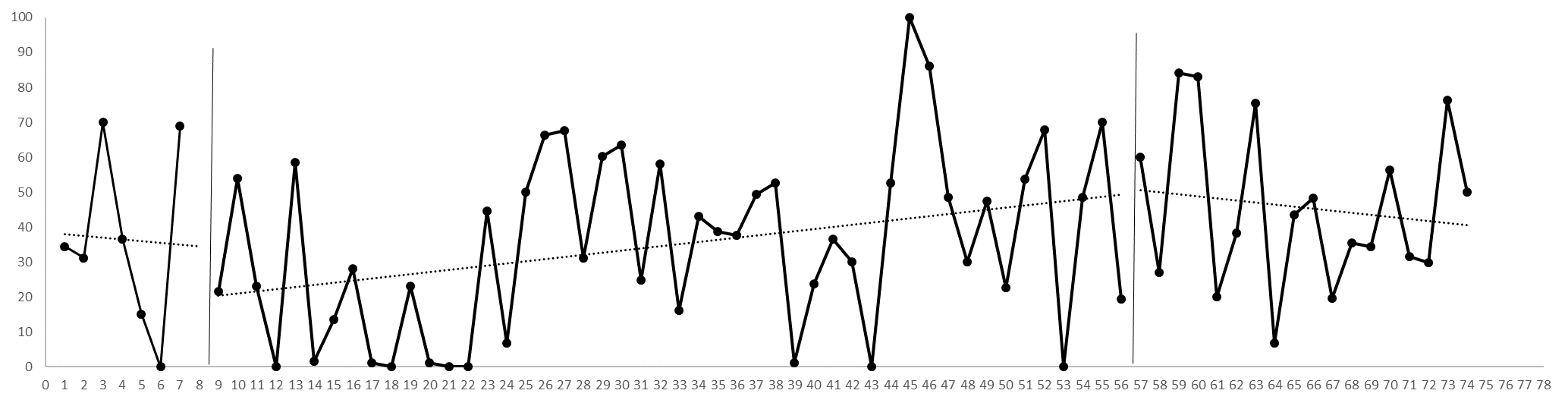


S10


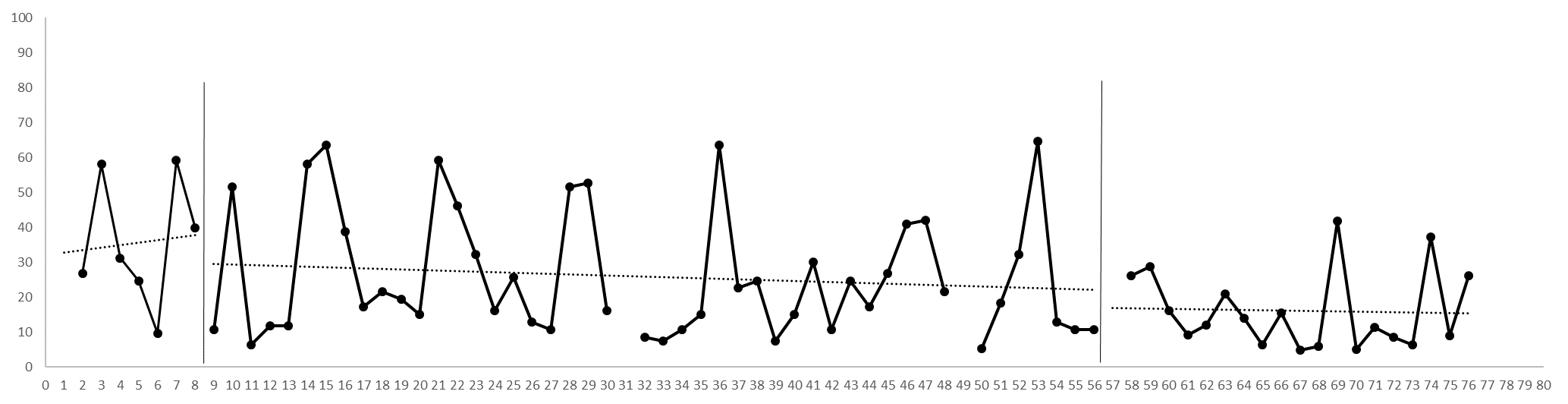


S2. (2) Raw data and trend for participants' behavioral avoidance ratings.

S01


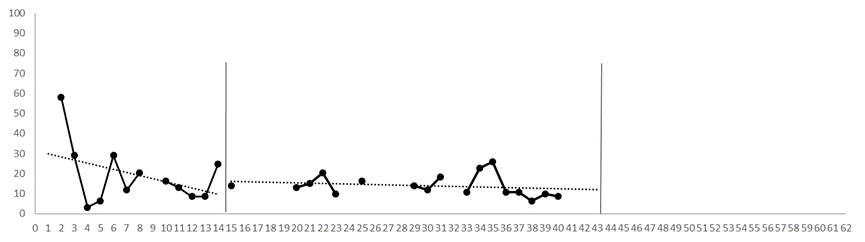


S05


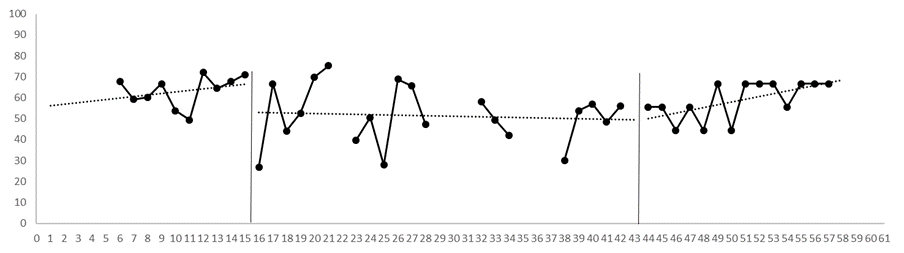


S08


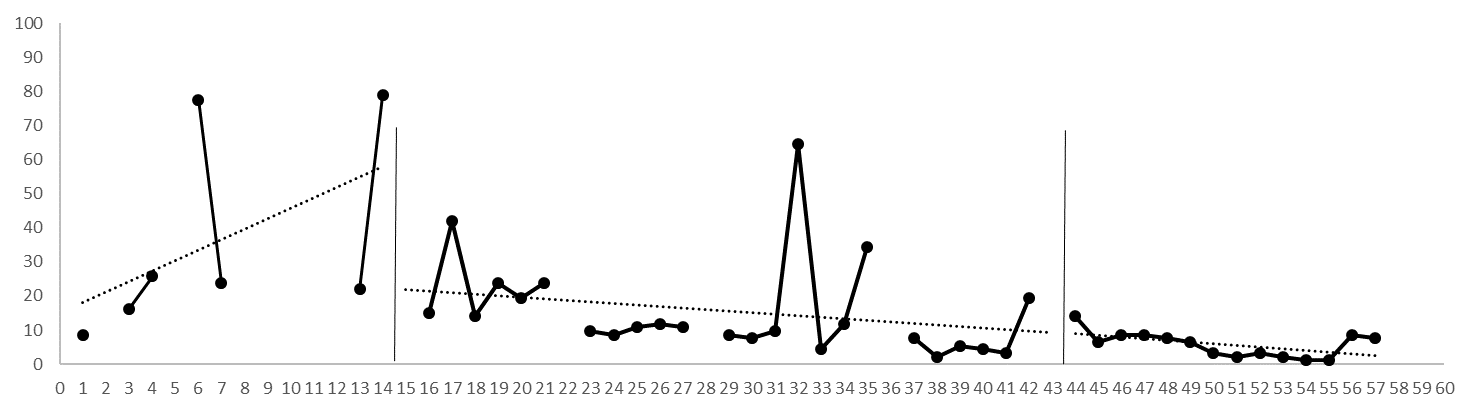


S02


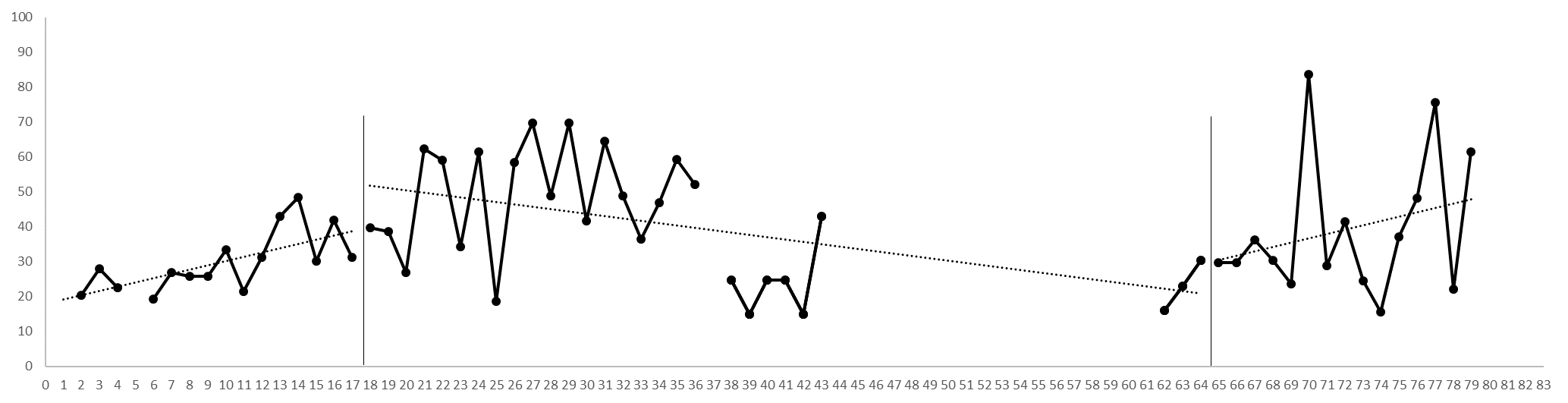


S06


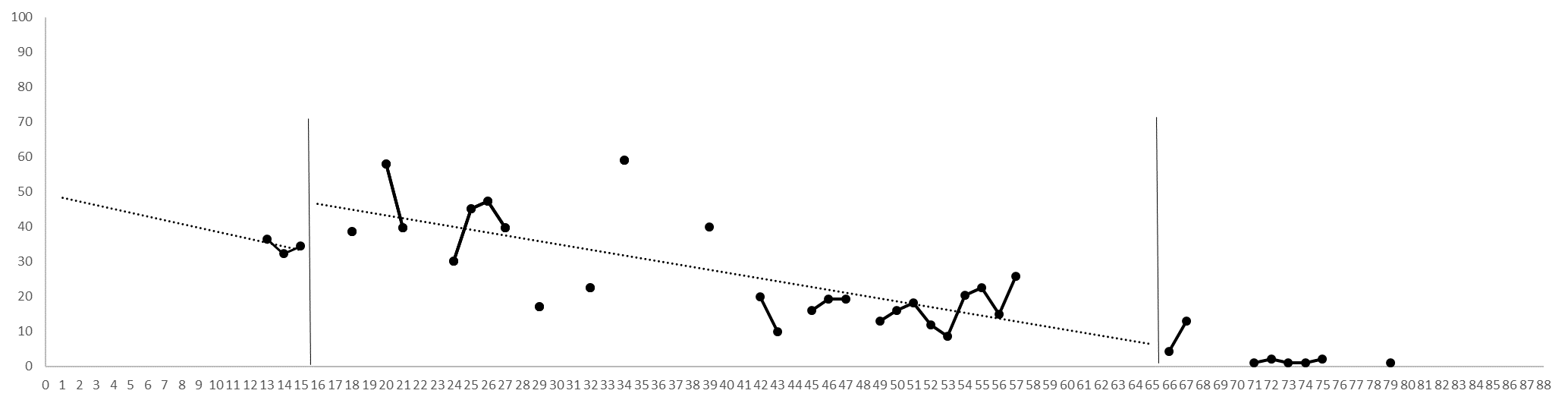


S09


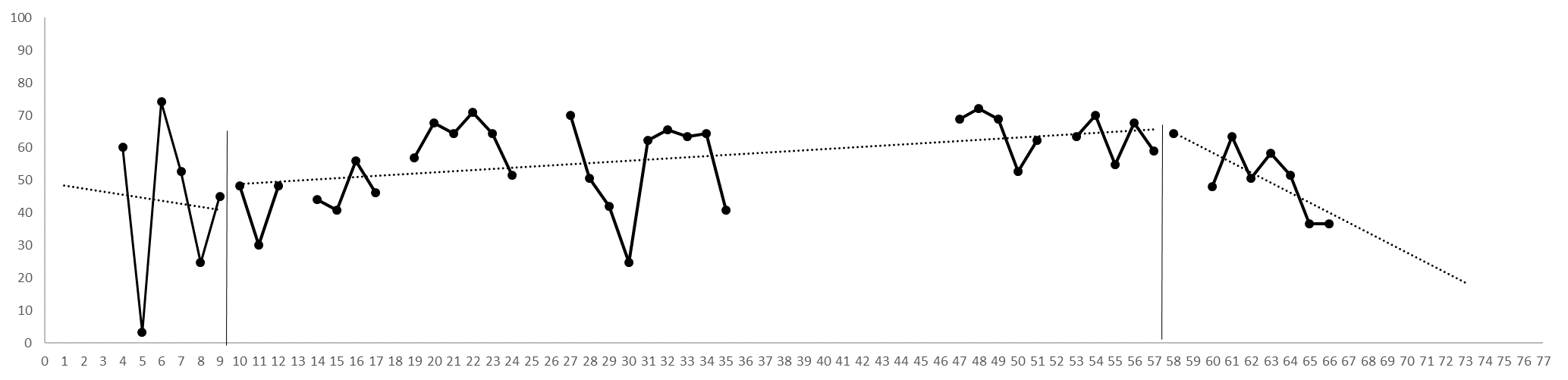


S04


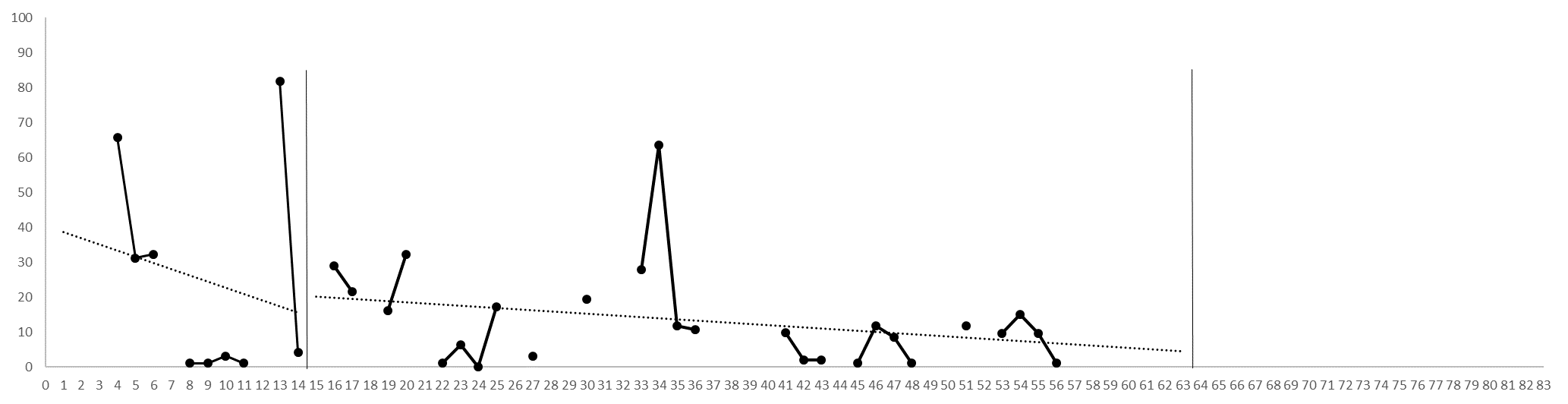


S07


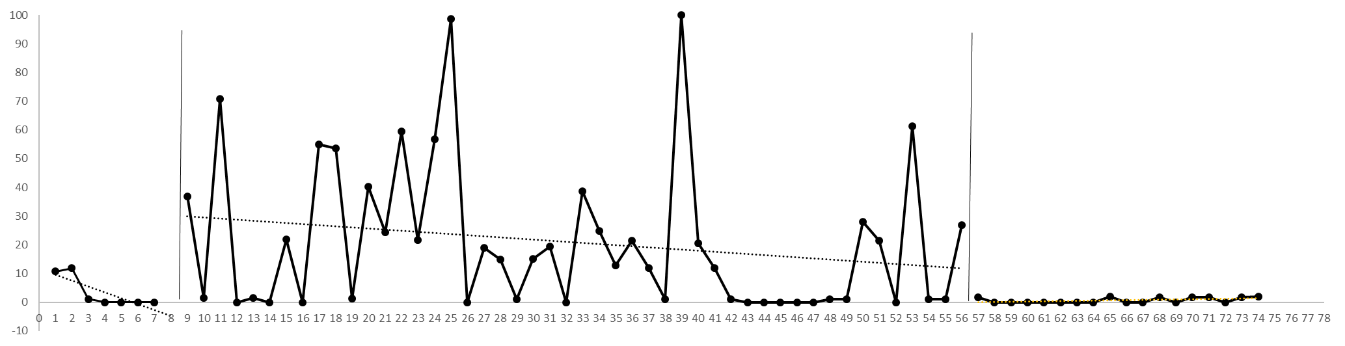


S10


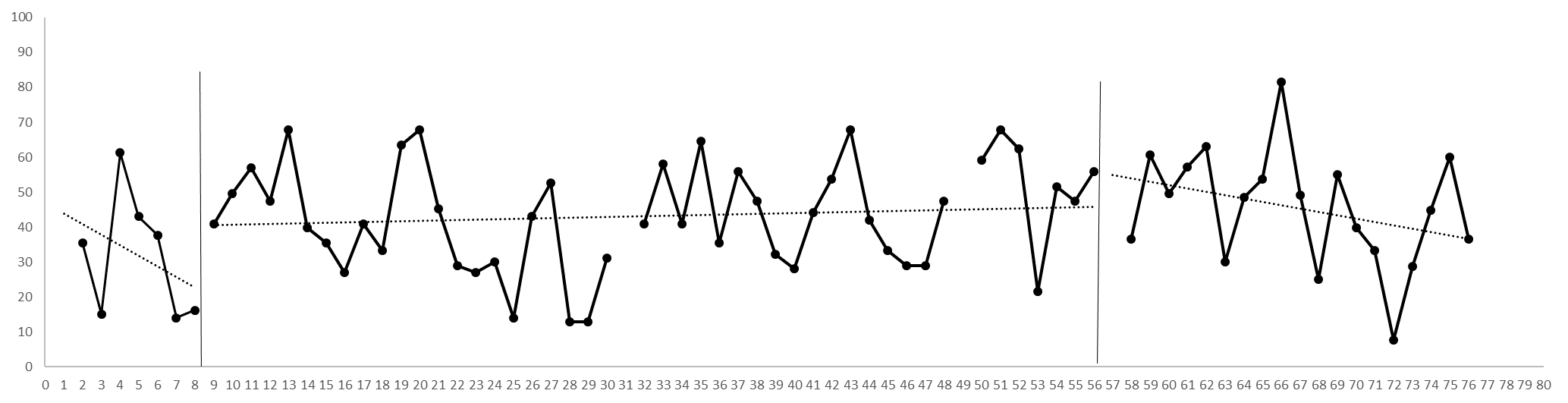


S2. (3) Raw data and trend for participants' self-focused attention ratings.

S01


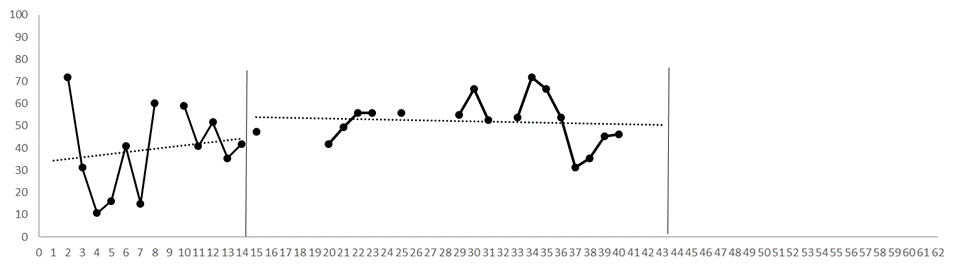


S05


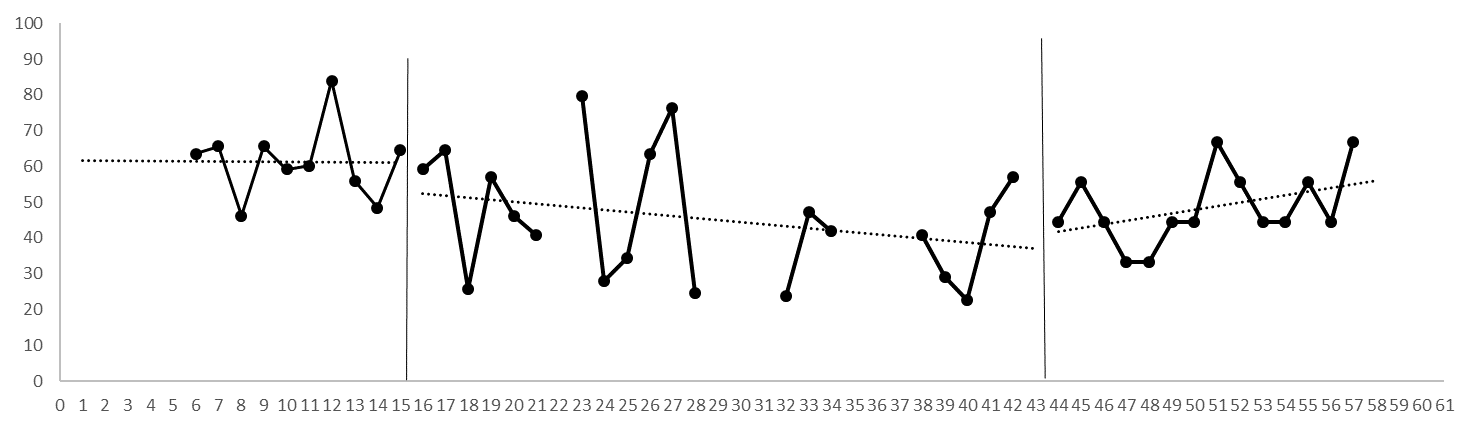


S08


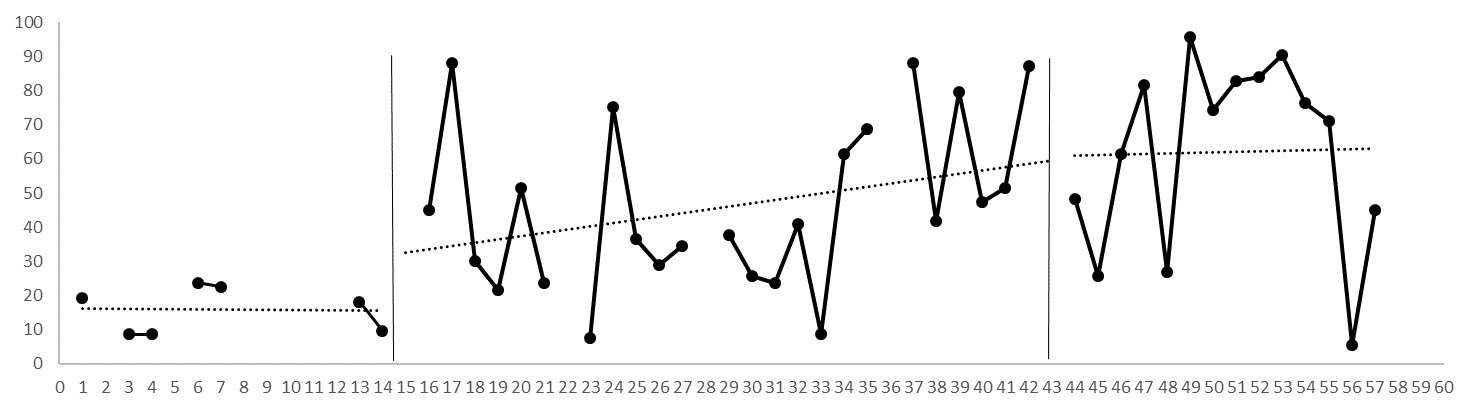


S02


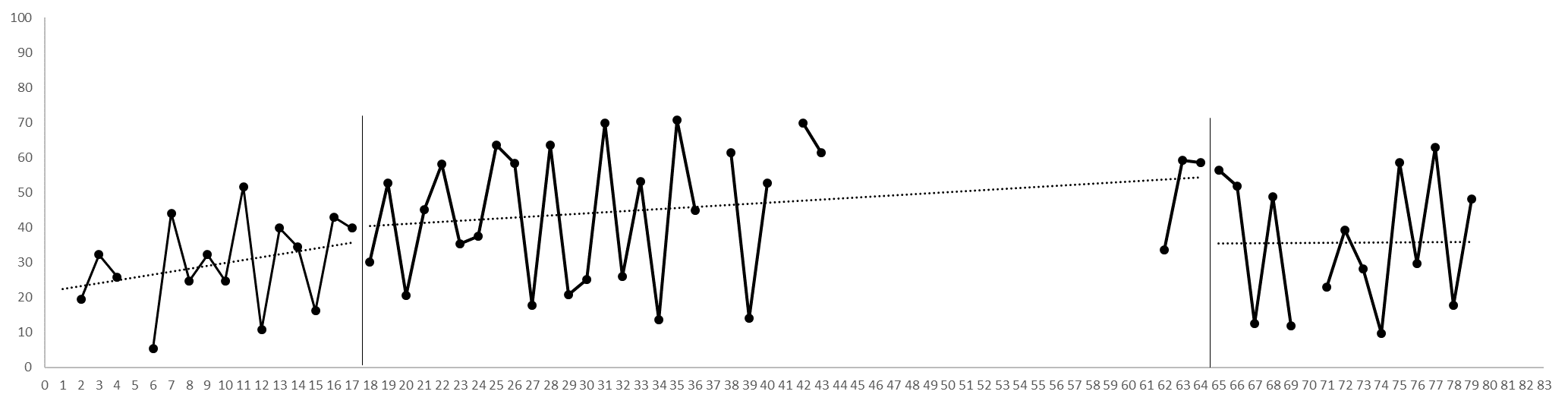


S06


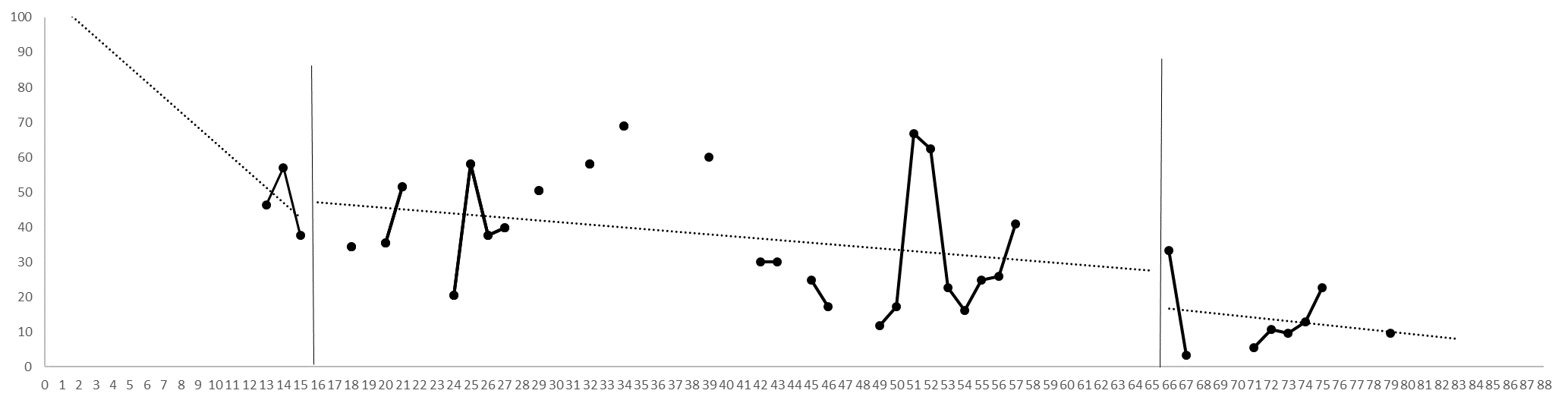


S09


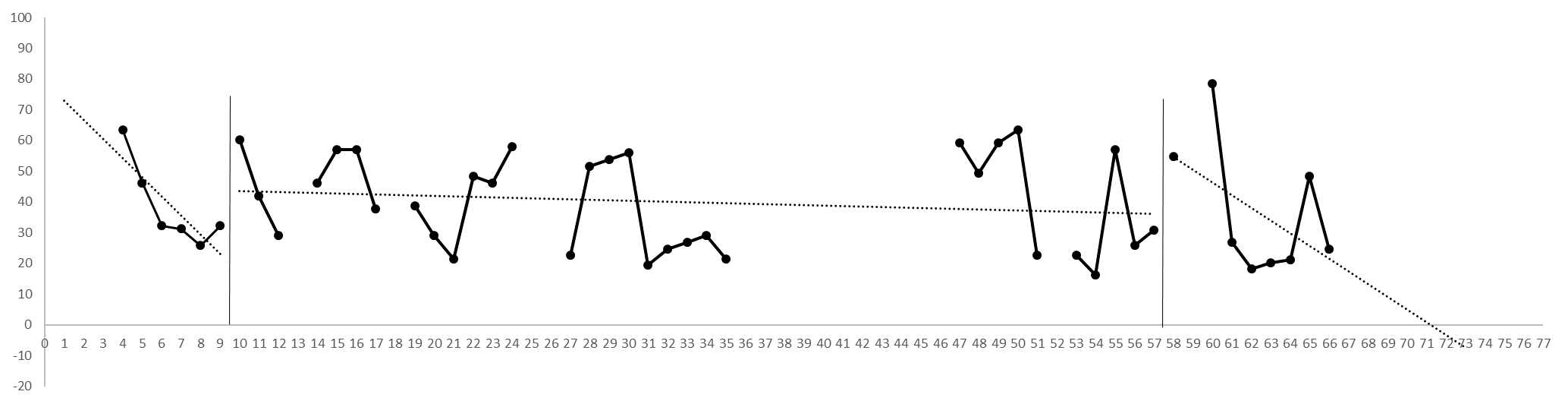


S04


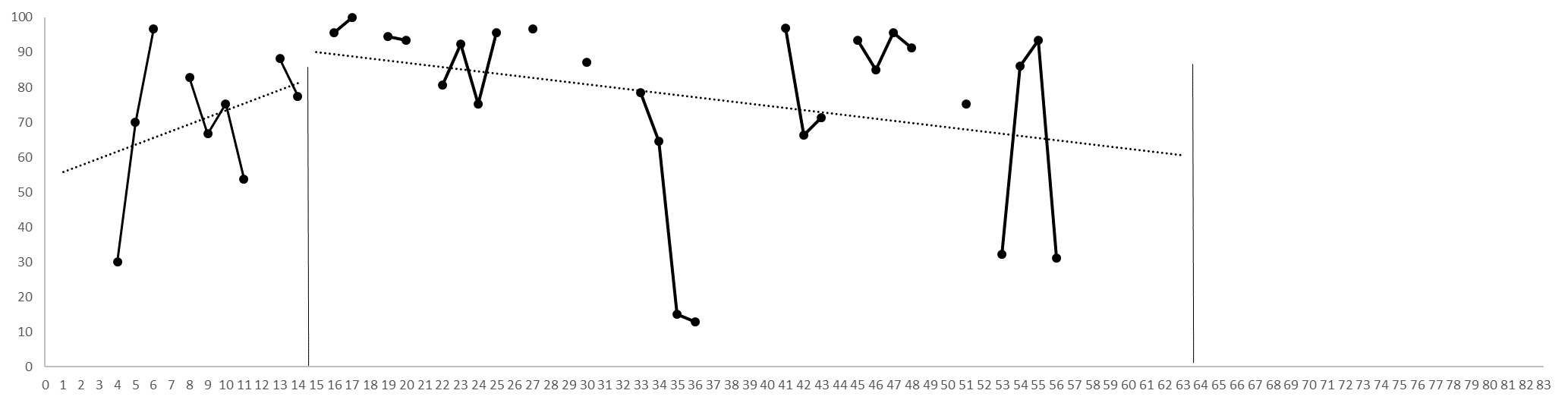


S07


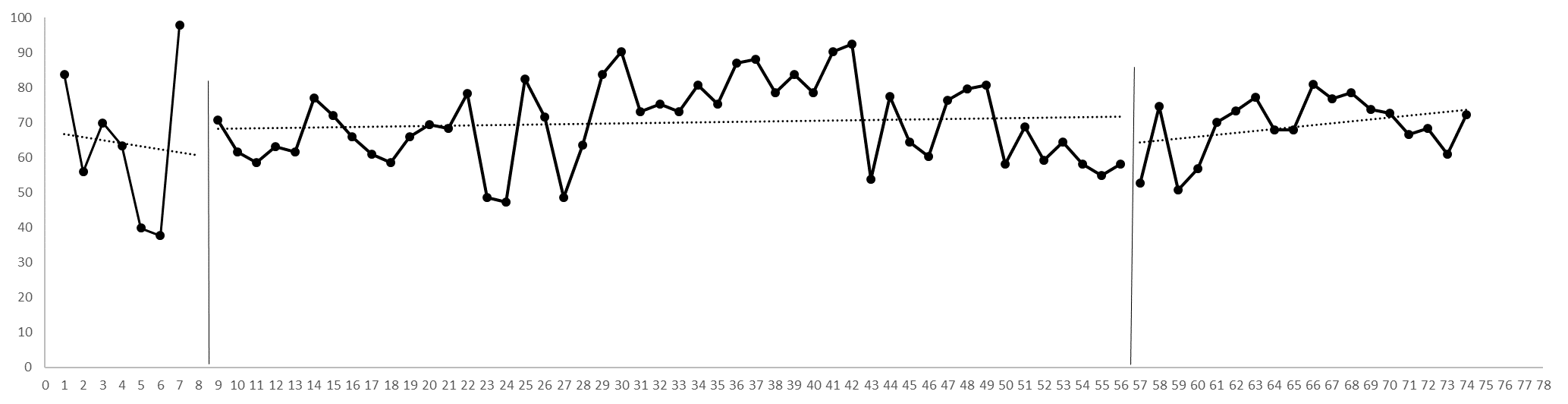


S10


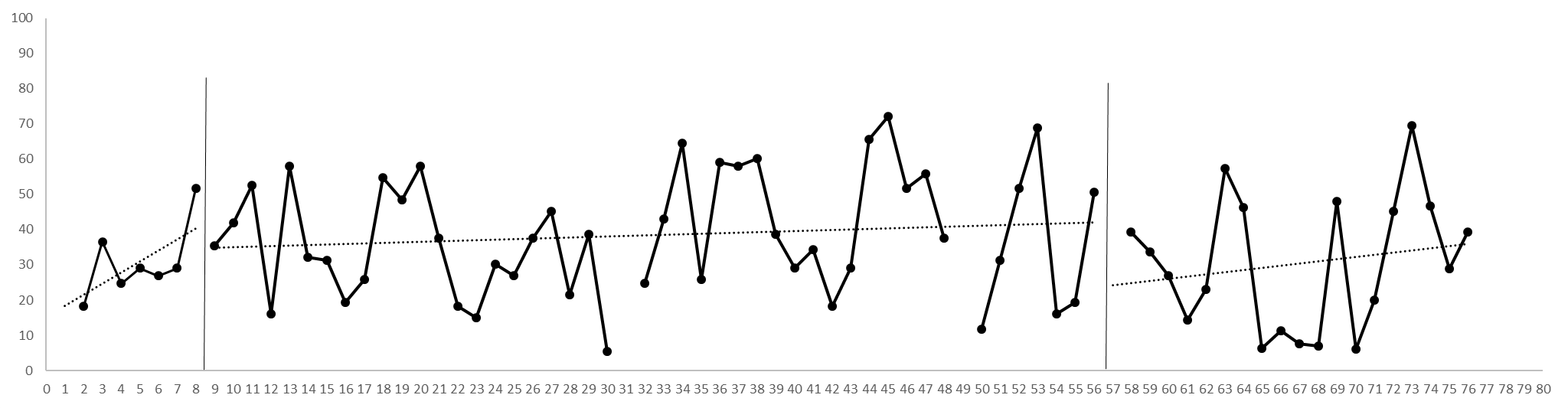


S2. (3) Raw data and trend for participants' rumination ratings.

| Table S3. Means, Standard deviation, TAU-U-and Cohen’s d scores of daily measures for participants of Condition 2 (ATT-BATD) | | | | | | | |
| --- | --- | --- | --- | --- | --- | --- | --- |
|  | *S02* | *S06* | *S09* |  | *S02* | *S06* | *S09* |
| ***Behavioral Activation*** | |  |  | ***Behavioral Avoidance*** | |  |  |
| *Mean A (SD)* | 37.85  (14.88) | 54.12  (9.39) | 28.85  (17.99) | *Mean A (SD)* | 29.96  (8.64) | 34.41  (2.15) | 43.37  (25.62) |
| *Mean ATT (SD)* | 53.03  (16.84) | 55.48  (13.27) | 39.52  (11.23) | *Mean ATT (SD)* | 49.39  (14.47) | 39.78  (13.72) | 53.29  (11.79) |
| *Mean BATD (SD)* | 46.66  (16.05) | 51.50  (9.74) | 34.52  (15.42) | *Mean BATD (SD)* | 24.09  (8.85) | 18.43  (7.60) | 60.07  (12.21) |
| *A-ATT d* | 1.02 | 0.14 | 0.59 | *A-ATT d* | 2.25 | 2.50 | 0.39 |
| *A-ATT TAU-U* | 0.44^*^ | -0.03 | 0.36 | *A-ATT TAU-U* | 0.55^*^(c)+ | 0.4 | 0.17 |
| *A-BATD d* | 0.59 | -0.28 | 0.32 | *A-BATD d* | -0.68 | -7.43 | 0.65 |
| *A-BATD TAU-U* | 0.33 | -0.13 | 0.15 | *A-BATD TAU-U* | 0.49^*^(c)+ | -0.87^*^ | 0.46 |
| *ATT-BATD d* | -0.38 | -0.30 | -0.45 | *ATT-BATD d* | -1.75 | -1.56 | 0.58 |
| *ATT-BATD TAU-U* | 0.18 | -0.47 | -0.16 | *ATT-BATD TAU-U* | 0.12 | -0.04 | 0.32 |
| **Self-focused attention** | | | | **Rumination** |  |  |  |
| *Mean A (SD)* | 29.61  (13.12) | 46.95  (9.70) | 38.53  (13.96) | *Mean A (SD)* | 32.54  (12.52) | 70.97  (6.72) | 38.71  (14.81) |
| *Mean ATT (SD)* | 42.44  (18.81) | 45.48  (14.41) | 43.68  (12.82) | *Mean ATT (SD)* | 47.59  (16.39) | 49.46  (19.53) | 44.89  (12.49) |
| *Mean BATD (SD)* | 51.31  (18.40) | 32.15  (18.24) | 36.48  (17.26) | *Mean BATD (SD)* | 23.76  (9.72) | 27.05  (11.70) | 51.50  (15.63) |
| *A-ATT d* | 0.98 | -0.15 | 0.37 | *A-ATT d* | 1.20 | -3.20 | 0.42 |
| *A-ATT TAU-U* | 0.4^*^+ | -0.03 | 0.21 | *A-ATT TAU-U* | 0.53^*^+ | -0.77^*^ | 0.23 |
| *A-BATD d* | 1.65 | -1.53 | -0.15 | *A-BATD d* | 0.59 | -6.54 | 0.64 |
| *A-BATD TAU-U* | 0.69^*^+ | -0.52 | -0.28 | *A-BATD TAU-U* | -0.40 | -1^*^ | 0.44 |
| *ATT-BATD d* | 0.47 | -0.93 | -0.56 | *ATT-BATD d* | -1.45 | -0.30 | -0.45 |
| *ATT-BATD TAU-U* | 0.09 | -0.64^*^ (c) | -0.03 | *ATT-BATD TAU-U* | -0.81^***^ | -0.67^*^ | 0.26 |
| *Note*. Funct. Impair. = functioning impairment; *d* = Cohen *d* score; TAU = Tau for non-overlap with baseline trend control; + = significant deterioration, (c) = corrected for baseline trend; ^*^ *p* < .05, ^***^ *p* <.001. | | | | | | | |

S4. Raw data and trend for participants' S02-Condition 2

Behavioral Activation


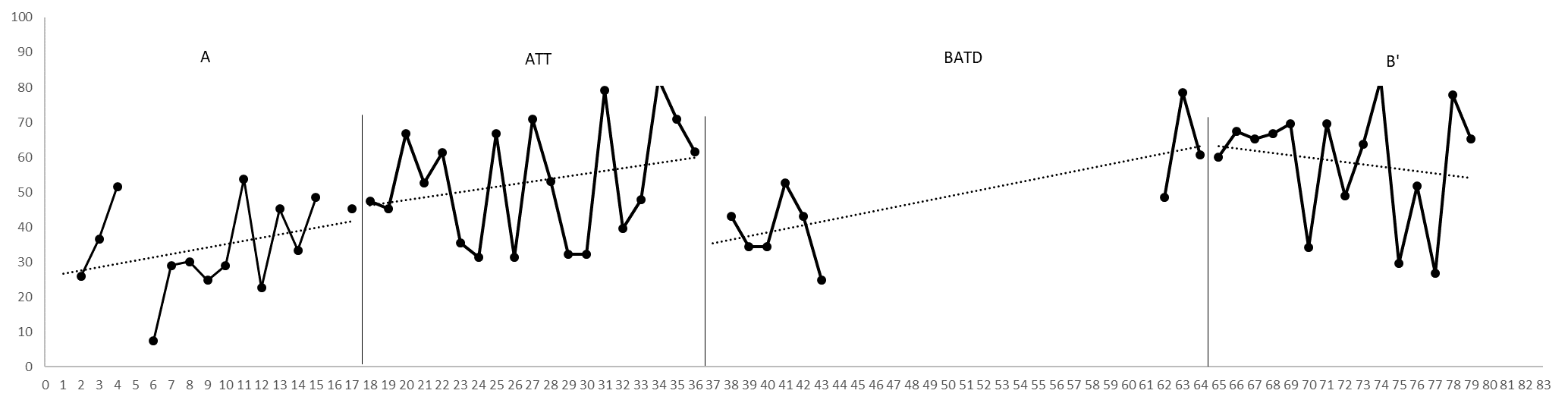


Behavioral Avoidance


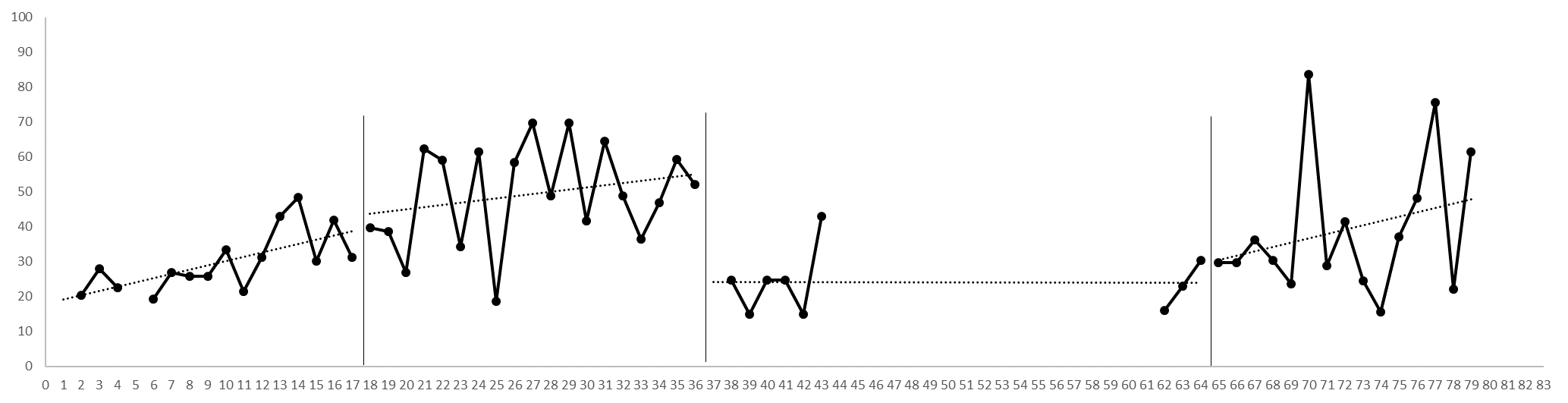


Self-focused attention


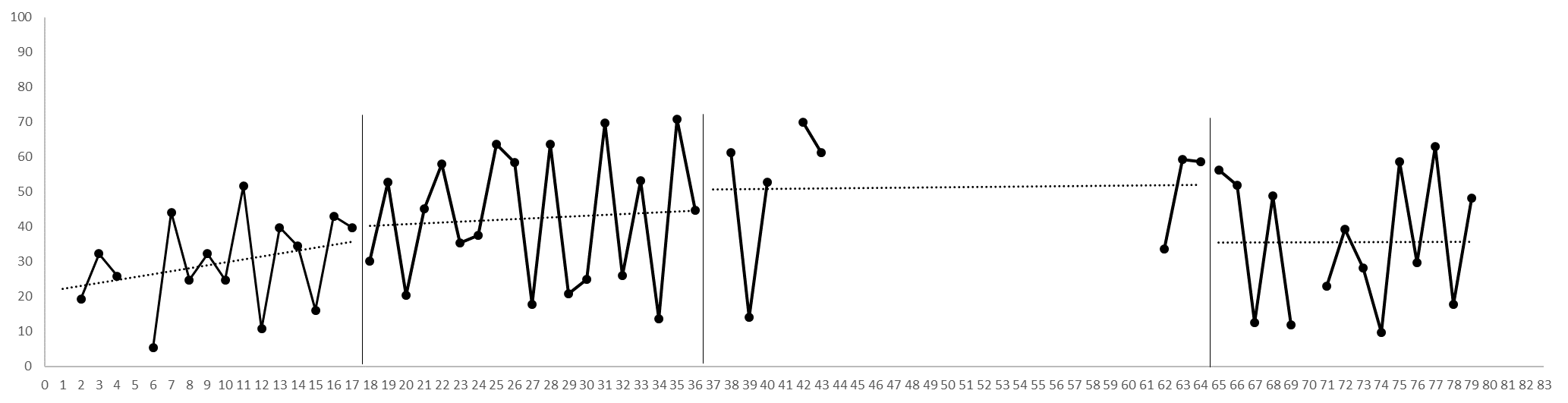


Rumination

| 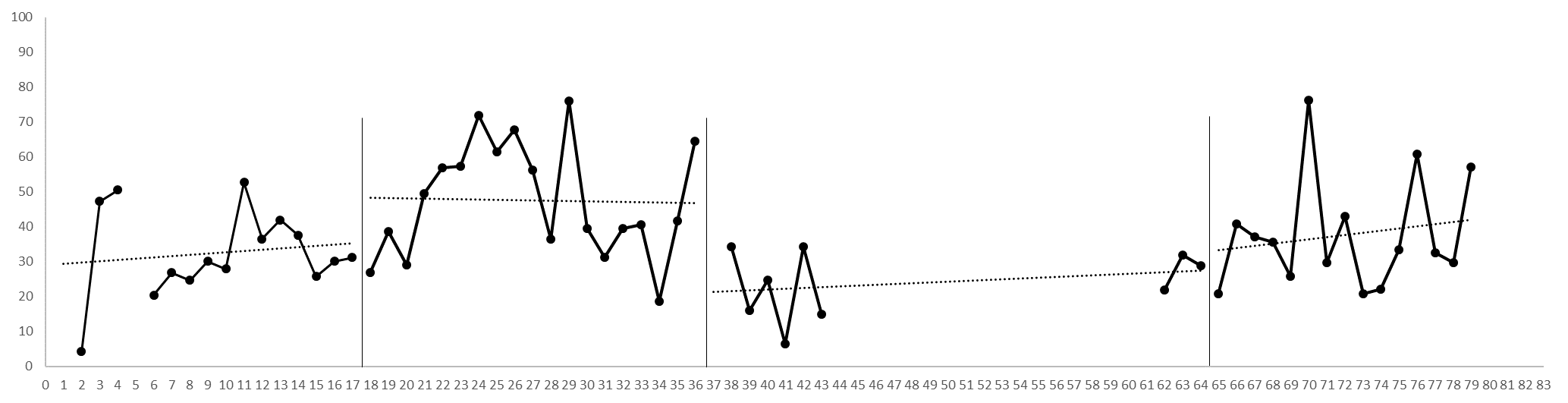 |
| --- |
|  |
|  |
|  |

S4. Raw data and trend for participants' S06-Condition 2

Behavioral Activation


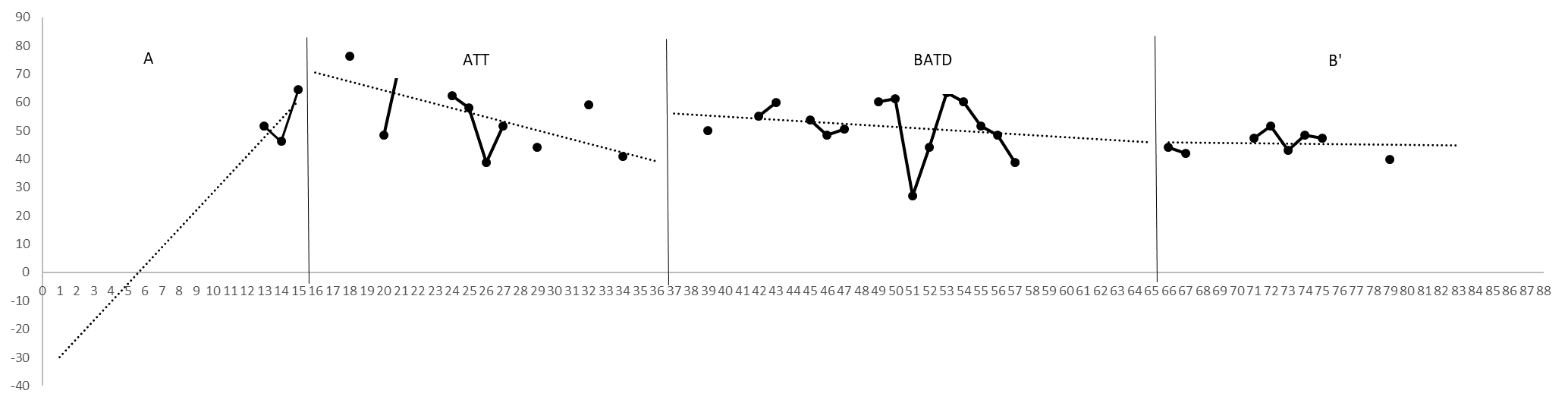


Behavioral Avoidance


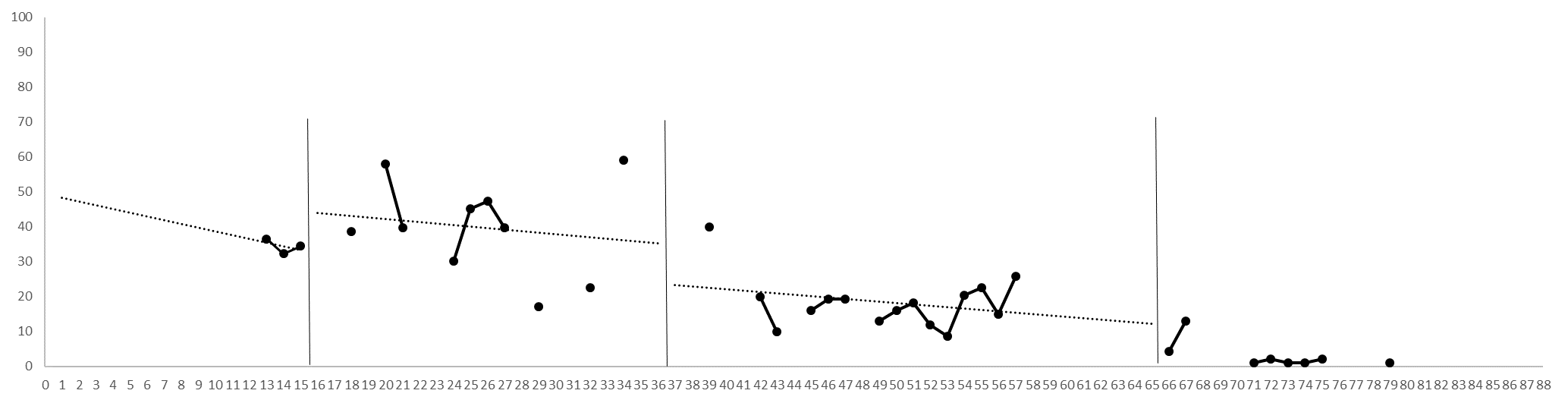


Self-focused attention


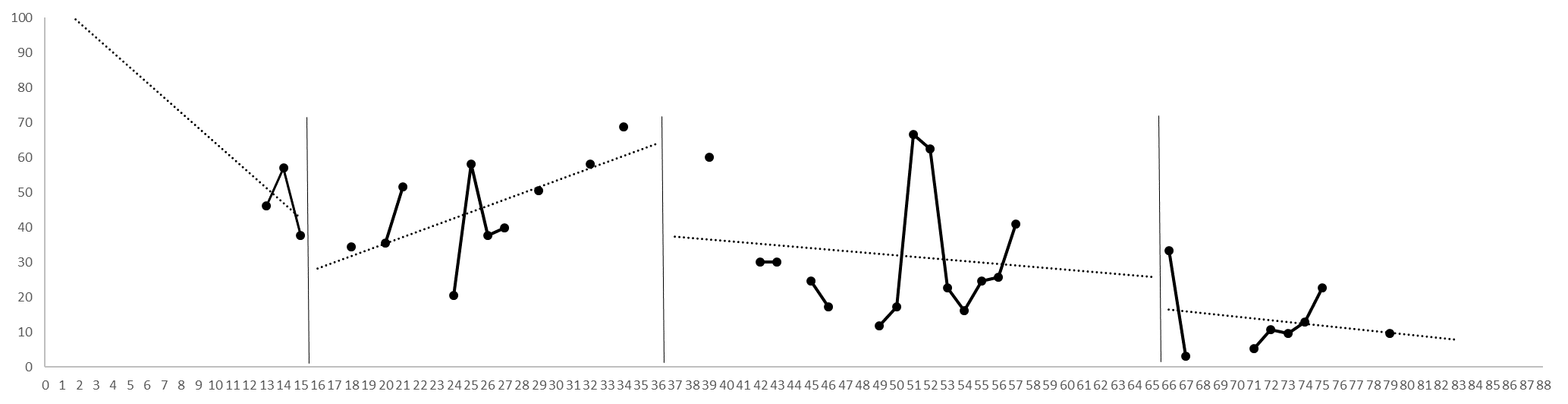


Rumination

| 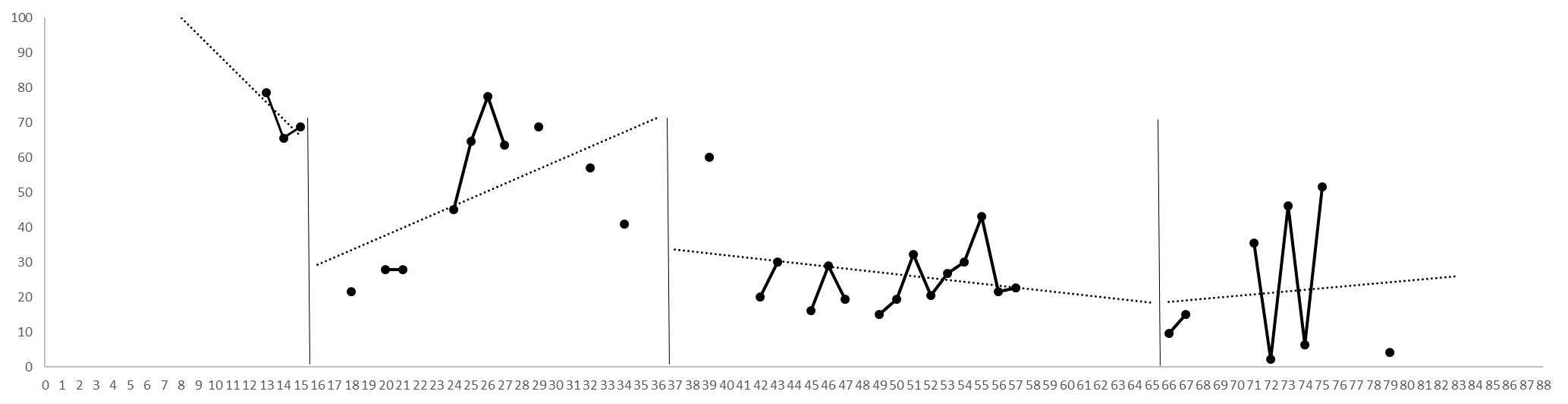 |
| --- |
|  |
|  |
|  |
|  |

S4. Raw data and trend for participants' S09-Condition 2

| Behavioral Activation  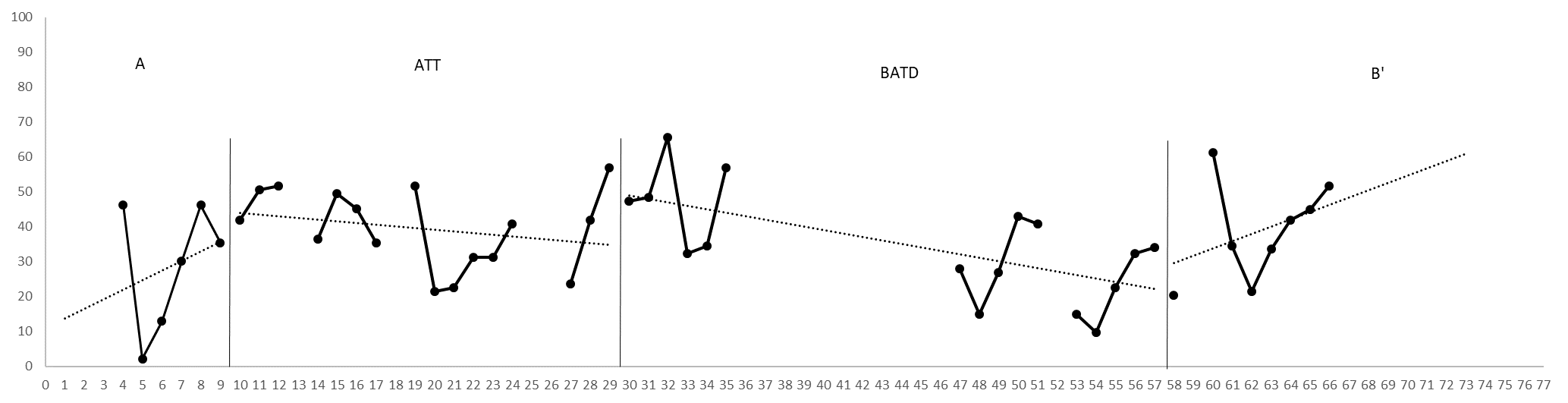  Behavioral Avoidance  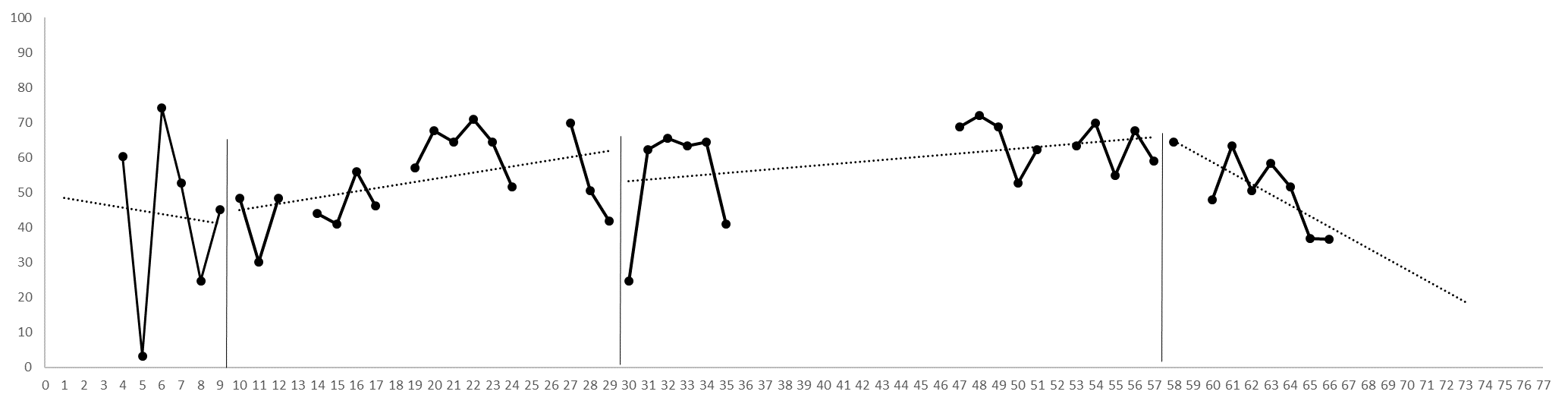  Self-focused attention  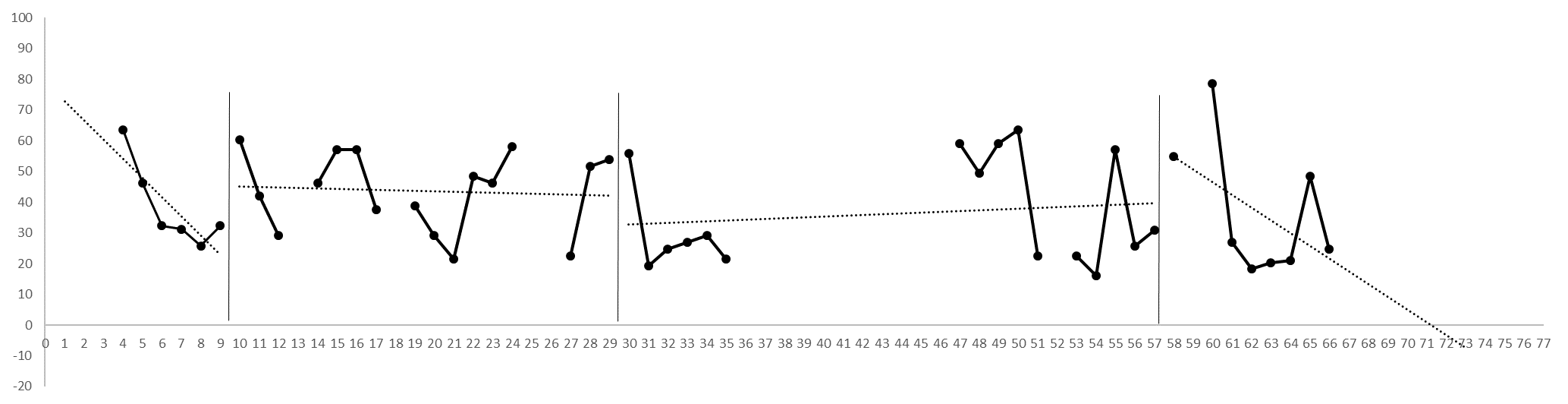  Rumination |
| --- |
| 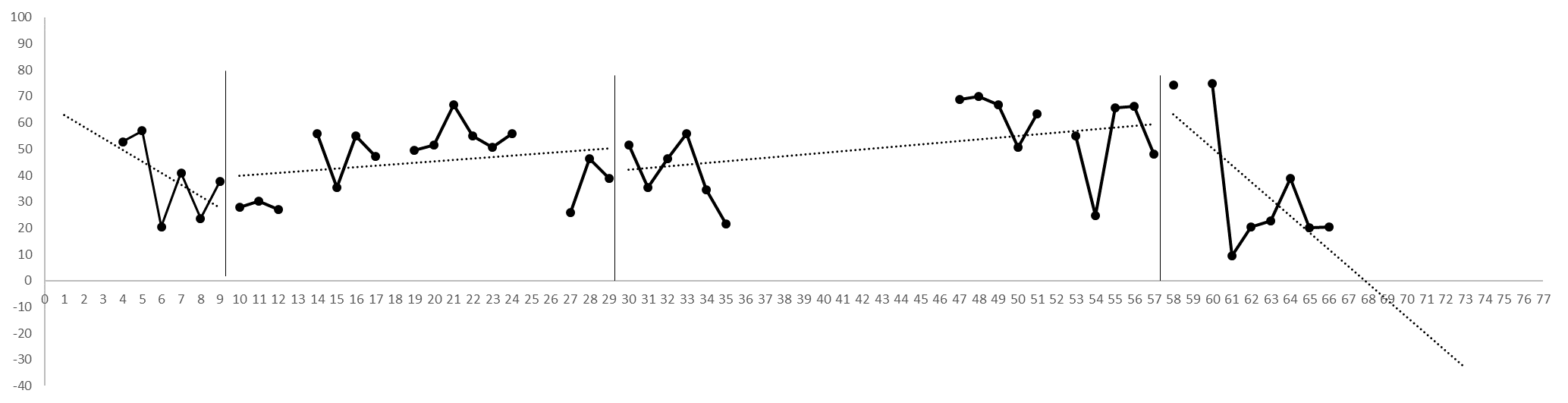 |
|  |
|  |
|  |

| Table S5. Means, Standard deviation, TAU-U-and Cohen’s d scores of daily measures for participants of Condition 3 (BATD-ATT) | | | | | | | |
| --- | --- | --- | --- | --- | --- | --- | --- |
|  | *S04* | *S07* | *S10* |  | *S04* | *S07* | *S10* |
| ***Behavioral Activation*** | |  |  | ***Behavioral Avoidance*** | |  |  |
| *Mean A (SD)* | 68.70  (32.15) | 36.56  (25.77) | 31.80  (17.73) | *Mean A (SD)* | 24.61  (30.77) | 3.38  (5.43) | 35.64  (18.08) |
| *Mean BATD (SD)* | 88.06  (11.64) | 29.48  (24.06) | 41.55  (15.76) | *Mean BATD (SD)* | 16.12  (15.97) | 25.53  (26.15) | 27.76  (19.59) |
| *Mean ATT (SD)* | 91.64  (9.84) | 41.78  (26.49) | 45.52  (14.24) | *Mean ATT (SD)* | 7.77  (5.34) | 14.75  (24.82) | 23.12  (14.91) |
| *A-BATD d* | 0.60 | -0.27 | 0.55 | *A-BATD d* | -0.28 | 4.08 | -0.44 |
| *A-BATD TAU-U* | 0.47^*^ | -0.19 | 0.29 | *A-BATD TAU-U* | -0.03 | 0.71^*^+ | -0.29 |
| *BATD-ATT d* | 0.31 | 0.51 | 0.25 | *BATD-ATT d* | -0.52 | -0.41 | -0.24 |
| *BATD-ATT TAU-U* | 0.33 | 0.23 | -0.09 | *BATD-ATT TAU-U* | -0.24 | -0.02 | -0.03 |
| ***Self-focused attention*** | |  |  | ***Rumination*** | |  |  |
| *Mean A (SD)* | 71.21  (19.83) | 64.06  (22.07) | 30.88  (10.66) | *Mean A (SD)* | 44.92  (40.74) | 35.33  (30.61) | 15.82  (12.03) |
| *Mean BA (SD)* | 77.50  (26.47) | 68.35  (10.82) | 36.64  (15.90) | *Mean BATD (SD)* | 41.07  (25.11) | 8.21  (12.35) | 27.92  (19.71) |
| *Mean ATT (SD)* | 75.99  (25.85) | 72.04  (12.82) | 41.22  (19.01) | *Mean ATT (SD)* | 46.48  (28.61) | 7.53  (8.85) | 30.05  (21.92) |
| *A-BATD d* | 0.32 | 0.19 | 0.54 | *A-BATD d* | -0.09 | -0.89 | 1.01 |
| *A-BATD TAU-U* | 0.27 | 0.18 | 0.24 | *A-BATD TAU-U* | 0.06 | -0.81^***^ | 0.45 |
| *BATD-ATT d* | -0.06 | 0.34 | 0.29 | *BATD-ATT d* | 0.22 | -0.06 | 0.11 |
| *BATD-ATT TAU-U* | 0.30(c) | -0.05(c) | 0.05 | *BATD-ATT TAU-U* | 0.09 | -0.02 | -0.09 |
| *Note*. Funct. Impair. = functioning impairment; *d* = Cohen *d* score; TAU = Tau for non-overlap with baseline trend control; + = significant deterioration, (c) = corrected for baseline trend; ^*^ *p* < .05, ^***^ *p* <.001. | | | | | | | |

S6. Raw data and trend for participants' S04-Condition 3

Behavioral Activation


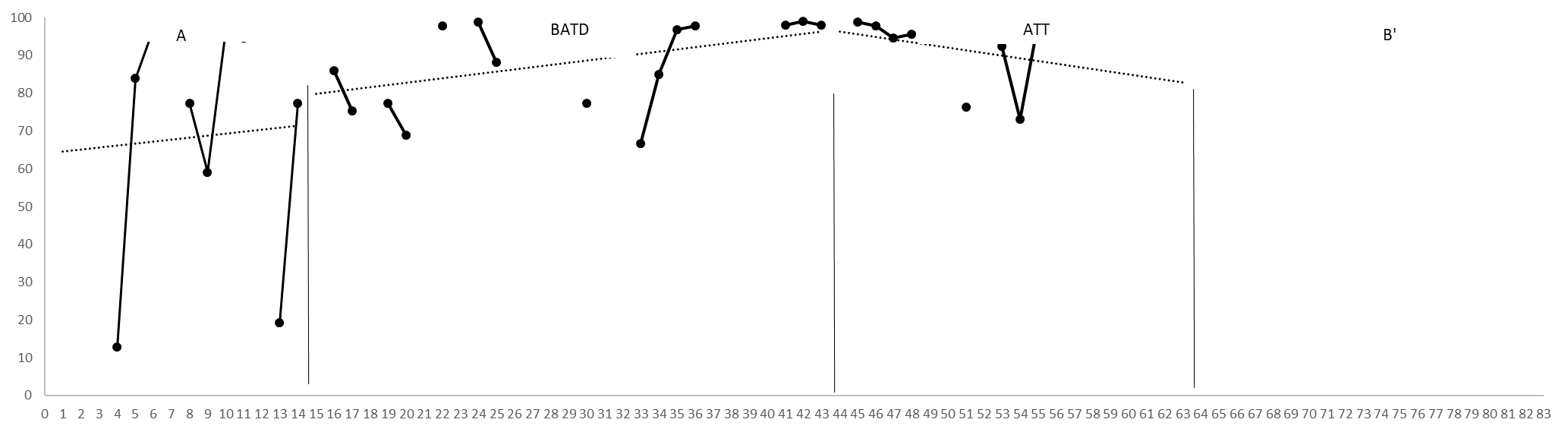


Behavioral Avoidance


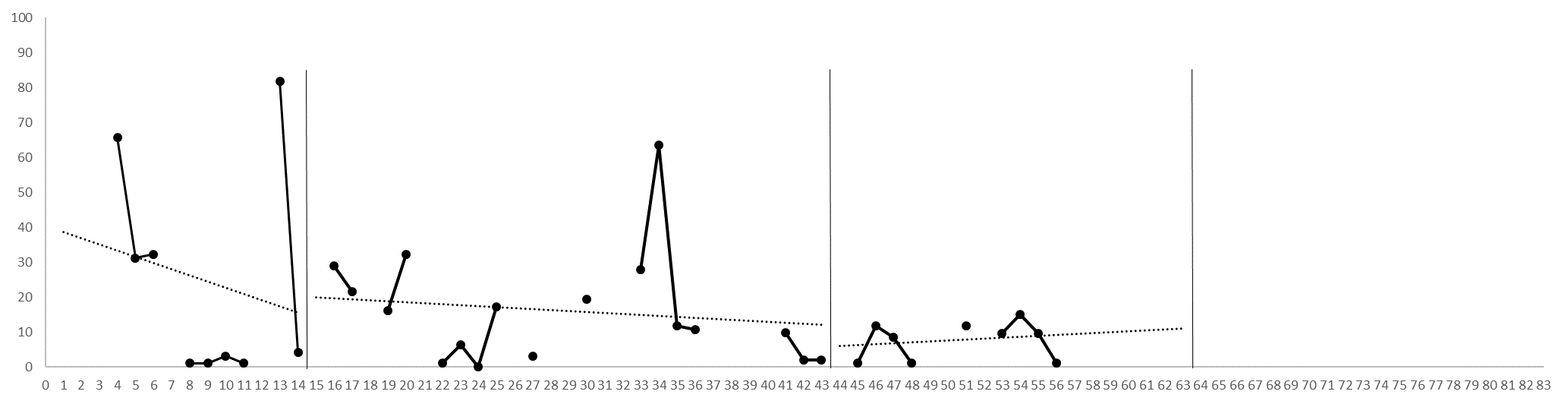


Self-focused attention


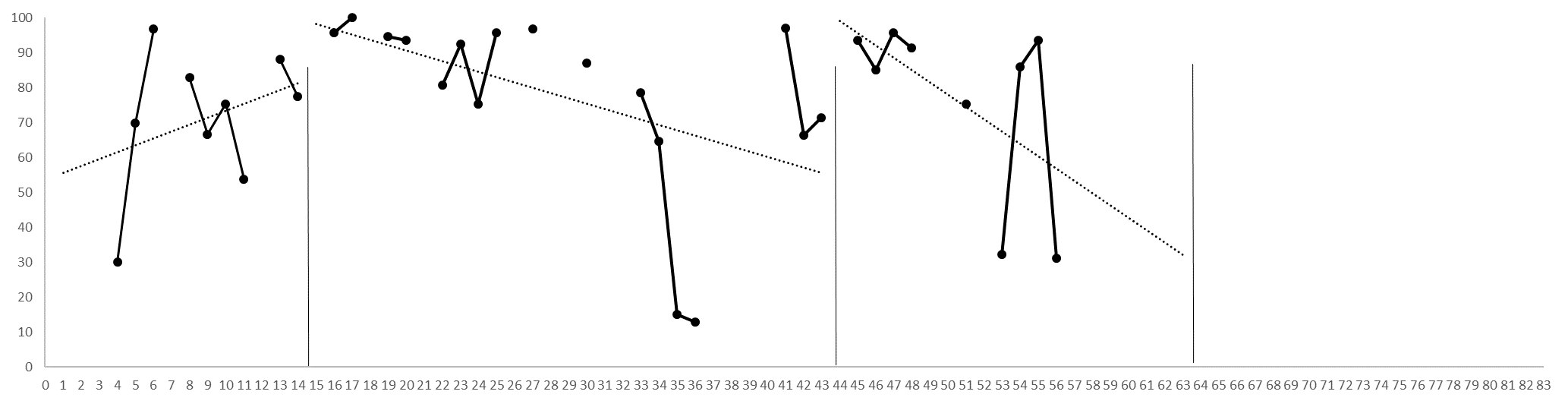


Rumination


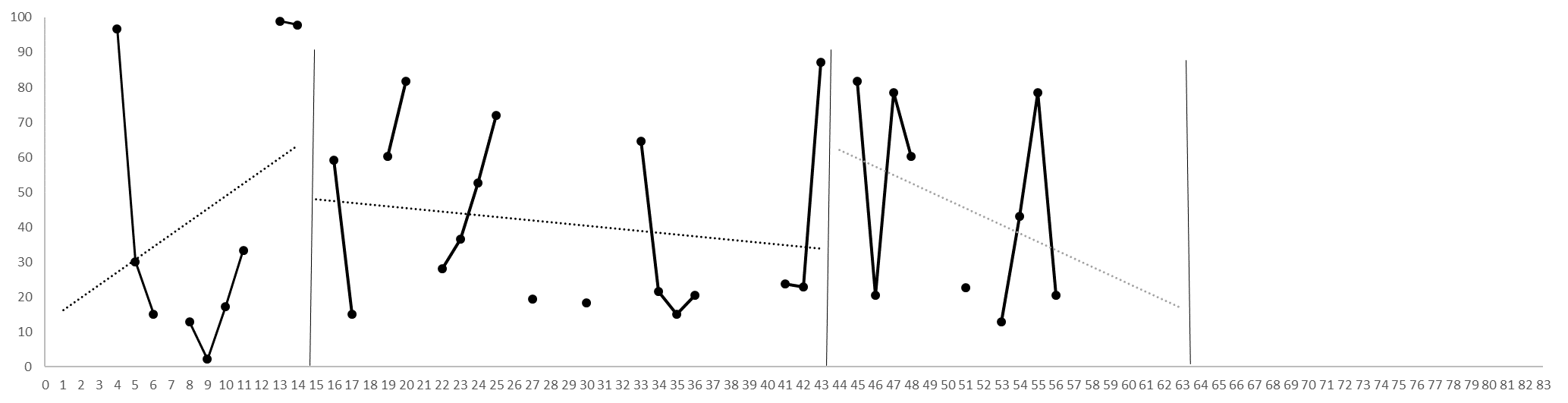


S6. Raw data and trend for participants' S07-Condition 3

Behavioral Activation


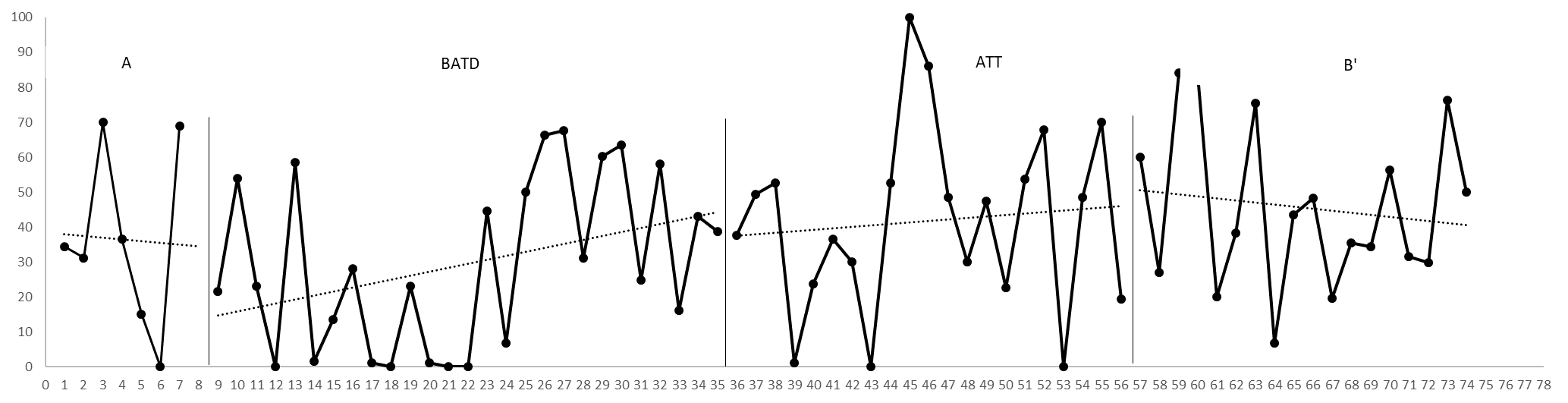


Behavioral Avoidance


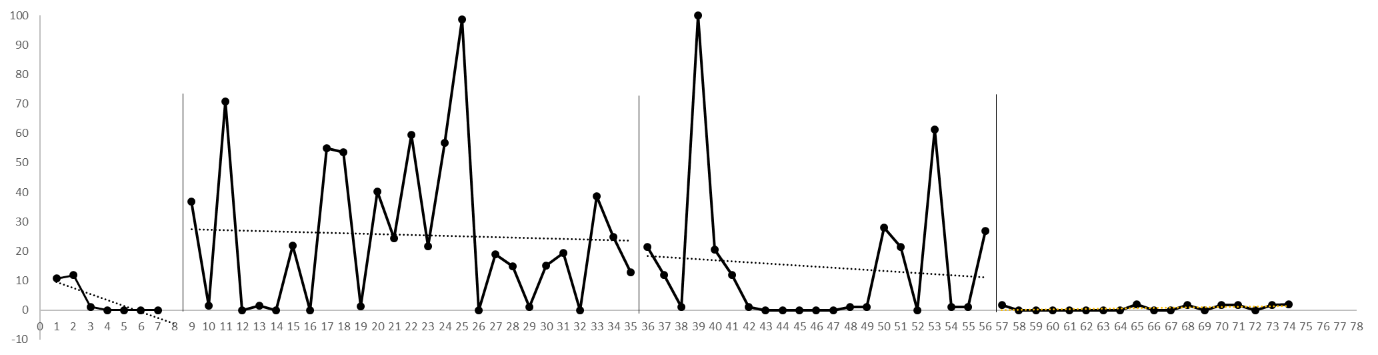


Self-focused attention


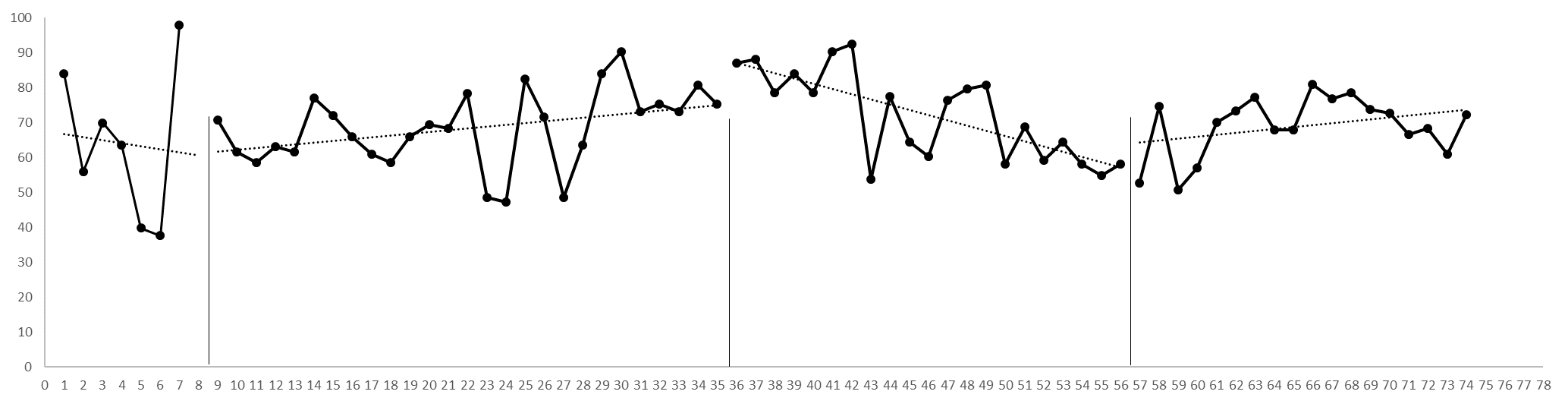


Rumination


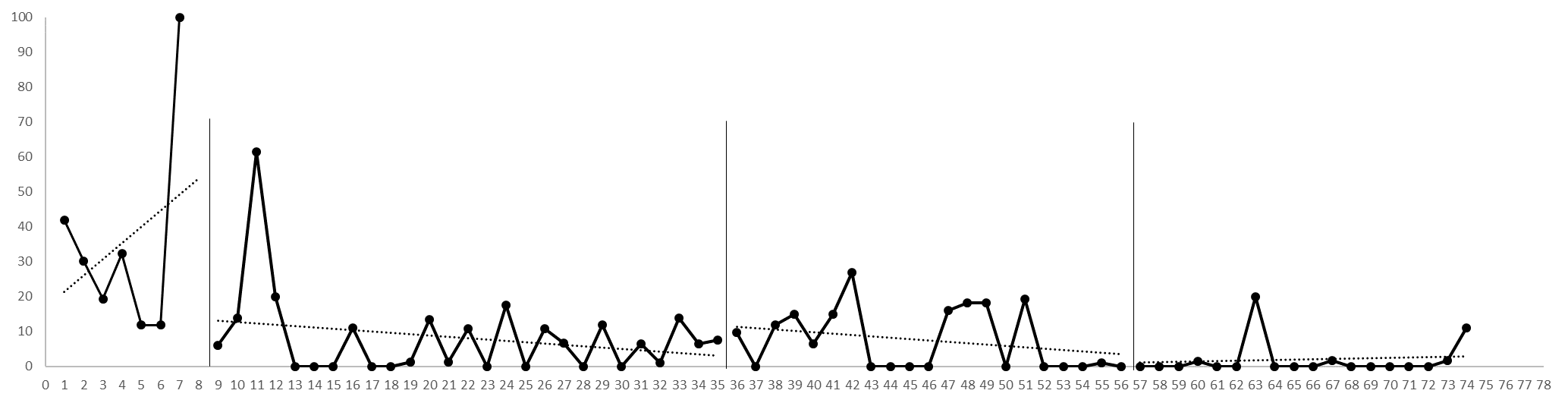


S6. Raw data and trend for participants' S10-Condition 3

Behavioral Activation


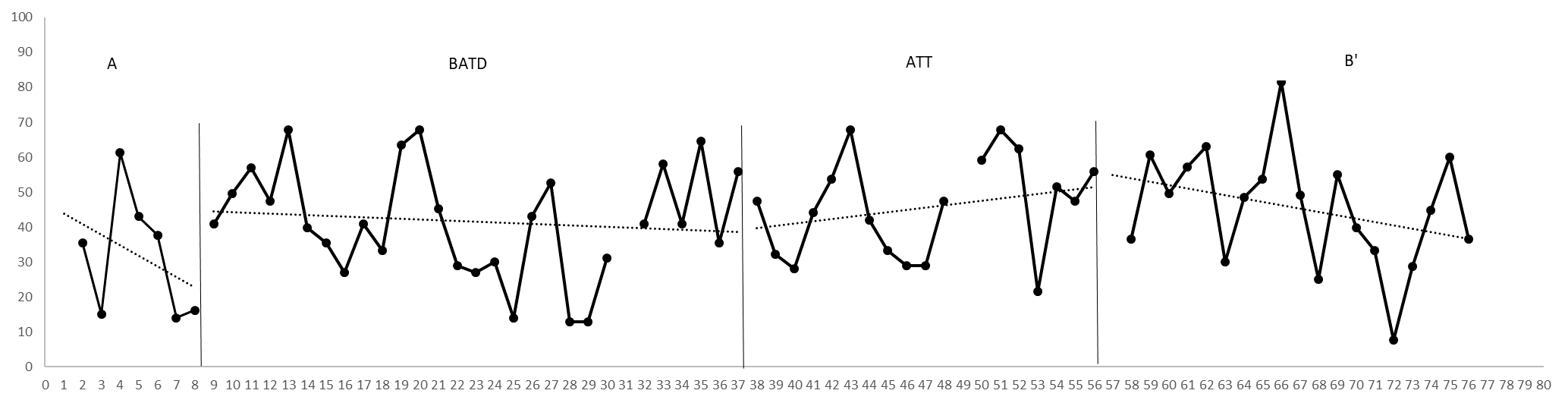


Behavioral Avoidance


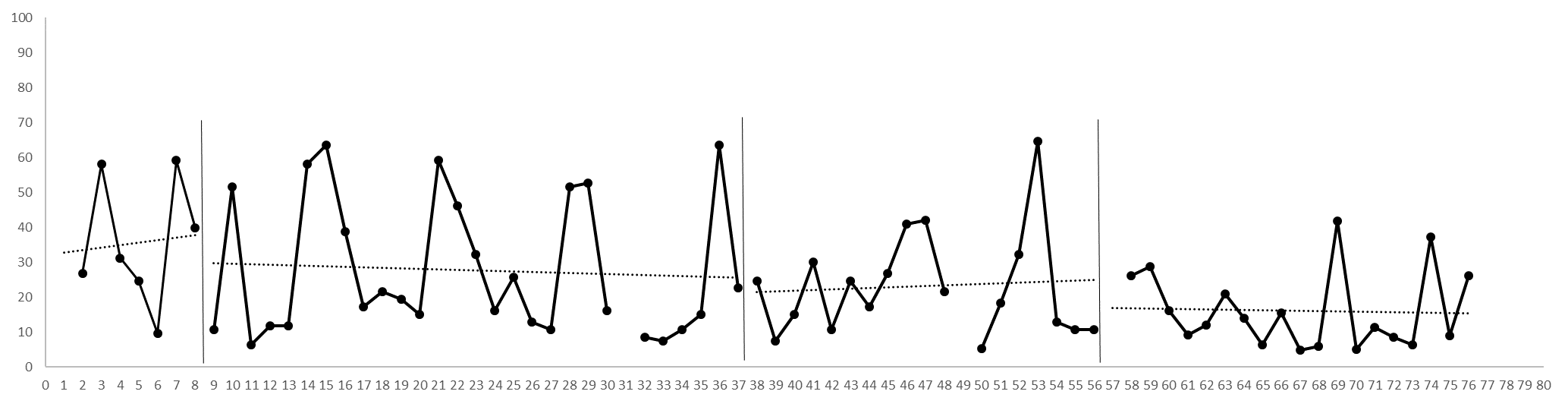


Self-focused attention


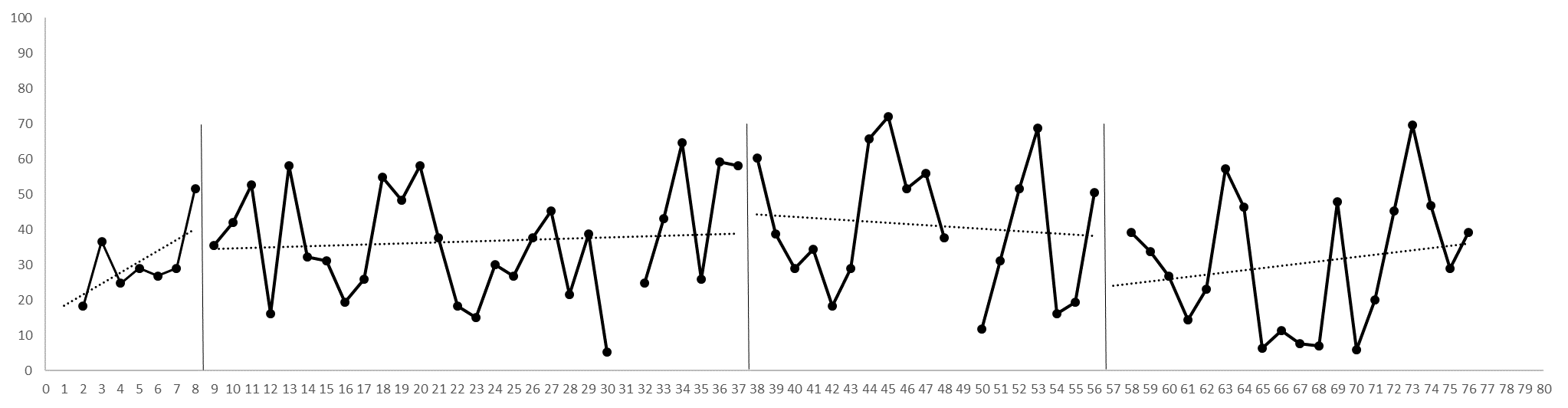


Rumination


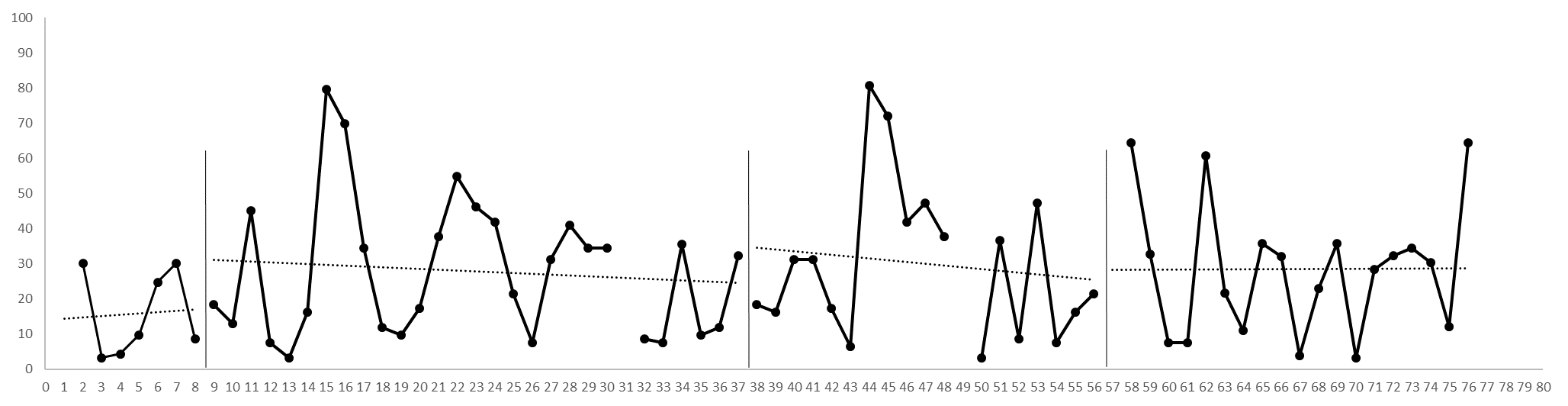

Supplement: Supplementary file 1 [file Data_Sheet_1.docx]
